# Metabolic activation of WHO-congeners PCB28, 52, and 101 by human CYP2A6: evidence from in vitro and in vivo experiments

**DOI:** 10.1007/s00204-024-03836-w

**Published:** 2024-08-13

**Authors:** Isabella Randerath, Thomas Schettgen, Julian Peter Müller, Jens Rengelshausen, Susanne Ziegler, Nathalia Quinete, Jens Bertram, Salah Laieb, Elke Schaeffeler, Andrea Kaifie, Katja S. Just, Aaron Voigt, Roman Tremmel, Matthias Schwab, Julia C. Stingl, Thomas Kraus, Patrick Ziegler

**Affiliations:** 1 Institute for Occupational, Social and Environmental Medicine, Medical Faculty, RWTH Aachen University, Pauwelsstrasse 30, 52074 Aachen, Germany; 2 Institute of Clinical Pharmacology, University Hospital of RWTH, 52074 Aachen, Germany; 3 Institute of Experimental Medicine and Systems Biology, RWTH Aachen University, Aachen, Germany; 4 Department of Chemistry and Biochemistry, Institute of Environment, Florida International University, 3000 NE 151s Street, North Miami, FL 33181 USA; 5 Dr. Margarete Fischer-Bosch-Institute of Clinical Pharmacology, Stuttgart and University of Tuebingen, Tübingen, Germany; 6 Department of Neurology, University Medical Center, RWTH Aachen University, 52074 Aachen, Germany; 7 Departments of Clinical Pharmacology, and Pharmacy and Biochemistry, University of Tuebingen, Tübingen, Germany

**Keywords:** Polychlorinated biphenyls, Cytochrome p450 monooxygenase, CYP2A6, Carcinogenesis

## Abstract

Despite extensive research on the metabolism of polychlorinated biphenyls (PCBs), knowledge gaps persist regarding their isoform-specific biotransformation pathways. This study aimed to elucidate the role of different cytochrome P450 enzymes in PCB metabolism, focusing on WHO-congeners 2,4,4′-trichlorobiphenyl (PCB28), 2,2′,5,5′-tetrachlorobiphenyl (PCB52), and 2,2′,4,5,5′-pentachlorobiphenyl (PCB101). Utilizing engineered HEK293 cell lines, we investigated the in vitro metabolism of these PCBs by CYP1A2, CYP2C8, CYP2C9, CYP3A4, CYP2A6, and CYP2E1, revealing robust production of hydroxylated metabolites. Our results show that CYP2A6 plays a major role in the metabolism of these congeners responsible for predominant formation of para-position hydroxylated metabolites, with concentrations reaching up to 1.61 µg/L (5,89 nM) for PCB28, 316.98 µg/L (1,03 µM) for PCB52, and 151.1 µg/L (441 nM) for PCB101 from a 20 µM parent PCB concentration. Moreover, concentration-dependent cytotoxic and cytostatic effects induced by reactive intermediates of the PCB hydroxylation pathway were observed in HEK293CYP2A6 cells, for all three congeners tested. CYP2A6 was specifically capable of activating PCBs 28 and 101 to genotoxic metabolites which produced genetic defects which were propagated to subsequent generations, potentially contributing to carcinogenesis. In a clinical study examining CYP2A6 enzyme activity in formerly exposed individuals with elevated internal PCB levels, a participant with increased enzyme activity showed a direct association between the phenotypic activity of CYP2A6 and the metabolism of PCB28, confirming the role of CYP2A6 in the in vivo metabolism of PCB28 also in humans. These results altogether reinforce the concept that CYP2A6 plays a pivotal role in PCB congener metabolism and suggest its significance in human health, particularly in the metabolism of lower chlorinated, volatile PCB congeners.

**Supplementary Information:**

The online version contains supplementary material available at 10.1007/s00204-024-03836-w.

## Introduction

Polychlorinated biphenyls (PCBs) are industrial chemicals that, when metabolically activated, can increase their toxicity and mutagenicity, potentially leading to DNA damage and an elevated risk of genetic mutations (Lauby-Secretan et al. 2016; Ludewig et al. 2008). However, our understanding of how this metabolic activation occurs in the human body, particularly in the liver, remains incomplete. The hydroxylation of PCBs during phase I metabolism is mediated by various isoforms of cytochrome P450 monooxygenases (CYPs) (Safe 1994), primarily expressed in the liver but also present in nearly all human tissues, localized in the inner mitochondrial membrane and smooth endoplasmic reticulum (Nelson et al. 1996). These enzymes exhibit significant genetic variability, with genetic polymorphisms identified across all major CYP isoenzymes involved in human drug metabolism (Zanger and Schwab 2013). Upon exposure to PCBs, the liver’s CYP system is induced, as demonstrated by feeding experiments in rats with commercial PCB mixtures or individual congeners, showing variable induction of CYP isoenzymes based on the position of chlorine atoms on the phenyl ring (Safe 1994). For example, non-ortho-chlorinated coplanar PCBs such as PCB126, PCB169, and PCB77 induce CYP1A1 and 1A2 expression, while ortho-chlorinated congeners such as PCB128, PCB153, PCB155, and PCB180 induce CYP2B1 (Parkinson et al. 1983). Structurally distinct PCBs such as PCB118, PCB138, and PCB170 induce the expression of both CYP1A1 and CYP2B (Parkinson et al. 1983). In phase I metabolism, theoretically, 837 monohydroxy metabolites can be formed from the existing 209 PCB congeners (Grimm et al. 2015). The formation of these metabolites strongly depends on the stereo- and regioselectivity of the catalyzing CYPs (Warner et al. 2009).

The volatile PCBs 2,4,4′-trichlorobiphenyl (PCB28), 2,2′,5,5′-tetrachlorobiphenyl (PCB52), and 2,2′,4,5,5′-pentachlorobiphenyl (PCB101) are found in the most prevalent Aroclor mixtures with differing mass proportions (PCB28: Aroclors 1016 > 1242 > 1248 > 1254 > 1260; PCB52: Aroclors 1248 > 1260 > 1016 > 1242 > 1254; PCB101: Aroclors 1254 > 1260 > 1248 > 1242 > 1016) (Ballschmiter and Zell 1980; Registry 2000). These PCBs, as well as the higher chlorinated congeners PCB138, PCB153, and PCB180, are categorized as indicator congeners (Beck and Mathar 1985). Whereas toxicological aspects were not considered in the definition of these indicator congeners, they are utilized as robust standards to differentiate diverse PCB profiles across varying sample matrices and are used for evaluating the risk posed by non-dioxin-like (NDL)-PCBs (EFSA 2005). While PCB138, PCB153, and PCB180 enter the human body mainly through diet, accounting for around 50% of the total PCB body burden (Anonymous 1999) PCB28, PCB52, and PCB101 are commonly found in polluted indoor air (Schettgen et al. 2012a; Gabrio et al. 2000; Hannah et al. 2022) and airborne PCBs in urban environments (Sun et al. 2006; Zhao et al. 2010; Marek et al. 2017; Liu et al. 2022). Moreover, PCB28, PCB52, and PCB101 have been found following occupational exposure, exemplified by individuals working in recycling companies, their family members and employees of surrounding companies (Schettgen et al. 2012b; Carpenter 2006). Importantly, past exposures to these congeners (as observed in former underground miners) can be estimated in retrospect by evaluating current PCB74 levels due to the comparatively long half-life of this congener (Esser et al. 2021b). Among PCB congeners, PCB28, PCB52, and PCB101 are more readily metabolized, resulting in the formation of characteristic OH-PCBs detectable using mass spectrometry (Quinete et al. 2015). Their metabolites exhibit significant differences in both half-lives and toxicity compared to each other and the parent compounds (Rengelshausen et al. 2023; Esser et al. 2021a; Safe 1994).

Although the toxicological aspects were not considered in the definition of these indicator congeners, the extent to which PCBs induce their own metabolism via CYP gene expression remains unclear, with genetic factors significantly influencing both the expression and activity of CYP enzymes (Zanger and Schwab 2013), thereby most likely affecting metabolic responses to PCB exposure. Studies have identified several CYP isoforms, including CYP2A6, CYP2B6, and CYP2E1, that are involved in the hydroxylation of various PCB congeners (Matsusue et al. 1996; McGraw and Waller 2006; Ariyoshi et al. 1995; Uwimana et al. 2019; Liu et al. 2017). These observations suggest that individuals exposed to PCBs have widely varying metabolic responses, influenced by the diverse congener composition and the specific CYP isoforms involved.

In our study, we aimed to investigate the specific role of cytochrome P450 enzymes, particularly CYP2A6, in the metabolism of key PCB congeners, namely PCB28, PCB52, and PCB101. By utilizing engineered HEK293 cell lines expressing various CYP isoforms, we sought to elucidate the pathways through which these enzymes biotransform PCBs into their hydroxylated metabolites. Furthermore, we explored the potential genotoxic effects of these metabolites, particularly focusing on the implications for human health and the propensity for genetic damage and carcinogenesis. Our results provide critical insights into the isoform-specific biotransformation of PCBs and highlight the significance of CYP2A6 in the metabolic activation of these environmental contaminants.

## Material and methods

### Analytical standards used in this study

The analytical standards of the PCB28-metabolites 2,4,4′-trichloro-5-biphenylol (5-OH-PCB28), its labeled analog ^13^C_6_-2,4,4′-trichloro-5-biphenylol (^13^C_6_-5-OH-PCB28), 2′,3′,4-trichloro-4′-biphenylol (4′-OH-PCB25) and 2,4′,5-trichloro-4- biphenylol (4-OH-PCB31) as well as the PCB52-metabolites 2,2′,5,5′-tetrachloro-3-biphenylol (3-OH-PCB52) and 2,2′,5,5′-tetrachloro-4-biphenylol (4-OH-PCB52) were custom synthesized at the Max Planck Institute for Biophysical Chemistry, Facility for Synthetic Chemistry (Göttingen, Germany). The characterization was performed via NMR spectroscopy and mass spectrometry (data not shown). 2,4,4′-Trichloro- 3′-biphenylol (3′-OH-PCB28) was purchased from Combi-Blocks (San Diego, CA, USA). 2,2′,4,5,5′-Pentachloro-4′-biphenylol (4′-OH-PCB101) and 2,2′,4,5,5′-pentachloro-3′-biphenylol (3′-OH-PCB101) were purchased by AccuStandard (New Haven, CT, USA). 2,2′,4,4′,5,5′-Hexachloro-3-biphenylol (3-OH-PCB153) was purchased from Wellington Laboratories (Guelph, Ontario, Canada). PCB 28 (2,4,4′-Trichlorobiphenyl), PCB 52 (2,2′,5,5′-Tetrachlorobiphenyl) and PCB 101 (2,2′,4,5,5′-Pentachlorobiphenyl) were purchased from LGC Standards (Augsburg, Germany).

### Generating CYP expressing transgenic HEK293 cell lines

For the generation of transgenic cell lines, we used semi-adherent HEK293 cells (DSMZ, ACC305, human embryonic kidney cells) which were cultured on a daily basis in DMEM medium supplemented with 10% (FCS) and 1% penicillin/streptomycin. In a first step CYP1A2, CYP2C8, CYP2C9, CYP3A4, CYP2A6, and CYP2E1 cDNA were cloned into the plasmid pcDNATM3.1/V5-His TOPO® using human liver RNA as a template and using the following primers: CYP1A2 *for*5′-TACAGATGGCATTGTCCCA-3′, CYP1A2 r*ev* 5′-GTTGATGGAGAAGCGCAG-3′; CYP2C8 *for* 5′-ACAATGGAACCTTTTGTGGTCC-3′, CYP2C8 rev 5′-GACAGGGATGAAGCAGATCTGG-3′; CYP2C9 *for* 5′-GAGAAGGCTTCAATGGATTC-3′, CYP2C9 *rev* 5′-GACAGGAATGAAGCACAG-3′; CYP3A4 *for* 5′-AGTAGTGATGGCTCTCATCCCAG-3′, CYP3A4 *rev* 5′-GGCTCCACTTACGGTGC-3′, CYP2A6 *for* 5′-ACCATGCTGGCCTCAGGGAT-3′, CYP2A6 *rev* 5′-GCGGGGCAGGAAGCTCAT-3′; CYP2E1 *for* 5′-CCCAGCGGCACCATGTCTG-3′, CYP2E1 *rev* 5′-TGAGCGGGGAATGACACAGA-3′. Final constructs were verified with Sanger DNA sequencing and restriction digestion. For transfection, 3 × 10^6^ HEK293 cells were pre-incubated in a 10 cm dish in DMEM medium with 10% FCS, but without antibiotics, for 18 to 24 h. When the cells reached approximately 70% confluency, they were transfected using TransIT-LT1 transfection reagent (Biozol, Eiching, Germany). The TransIT-LT1 reagent was warmed to room temperature and vortexed. 1.63 mL Opti-MEM, 16.25 µL plasmid DNA (1 µg/µL), and 48.75 µL TransIT-LT1 reagent were mixed and incubated for 15 to 30 min. The mixture was then added dropwise to the medium of the cells, and the cells were incubated again at 37 °C and 5% CO2. After 24 h, the medium was replaced with standard medium and geneticin was added for selection (1 mg/mL). Cells were selected for 6–8 weeks in bulk culture, and single cell clones were obtained subsequently by limiting dilution. Each clone was checked for insertion by PCR using genomic DNA as a template.

### Analysis of PCB metabolites in transgenic HEK293 cell lines

For the incubation of PCB28, PCB101, and PCB52 with CYP expressing transgenic cell lines, 4 × 10^5^ cells were cultured geneticin free in Dulbecco Modified Eagle Medium (DMEM) supplemented with 10% of FCS in a total volume of 2.5 mL. Parallel samples were run without the addition of PCBs. At a confluence of 80%, PCBs (20 μM each, dissolved in ethanol) were added and cells were incubated for 24 h before harvest of supernatants. Supernatants were diluted 1:2 with 80 μL acetate buffer 0.1 M (pH = 5.3). 100 μL of this dilution were further incubated with 100 μL of ammonium acetate buffer 0.1 M (pH = 5.3) and 5 μL of β-Glucuronidase/Arylsulfatase enzyme overnight in a drying oven at 37 °C for enzymatic hydrolysis to release conjugated compounds. 50 μL of a mix of internal standards (10 ng/mL) and 600 μL of methanol were added to the samples, then mixed by vortexing for 1 min and centrifuged for 10 min at 3400*g*. The individual supernatants were transferred to glass LC vials and evaporated to approximately 50 μL at 45 °C under a gentle stream of nitrogen. Finally, 0.1 mol/ L ammonium acetate buffer was added to a final volume of 100 μL and then transferred to an insert for analysis. The online solid-phase extraction (SPE) method coupled to liquid chromatography–tandem mass spectrometry was done as described previously using an API 5500 QTrap mass spectrometer (AB Sciex, Darmstadt, Germany) equipped with electrospray ionization (ESI) interface (Quinete et al. 2015).

### MTT assay

For the determination of metabolic activity, HEK293 cells as well as transgenic HEK293CYP2A6 and HEK293CYP2C9 cells were plated into 96-well flat-bottomed microtiter plates (Becton Dickinson, Heidelberg, Germany) at a density of 1 × 10^5^ cells in 100 μL of media. Cells were allowed to grow for 24 h geneticin free before PCBs were added. Controls included cells grown in medium with and without ethanol (maximal 1% (vol/vol)) added. After 48 h, the ability of remaining viable cells to transform 3-(4,5-dimethylthiazol-2-yl)-2,5-diphenyltetrazolium bromide (MTT) into formazan was assessed (MTT Cell Proliferation Assay, ATCC, Manassas, USA). The absorbance of the samples was measured on a Microplate Reader (FLUOstar, BMG Labtech, Ortenberg, Germany) at 450 nm.

### Comet assay

For neutral comet assays, transgenic CYP2A6 and transgenic CYP2C9 cells were treated with parent PCBs for 5 h. After treatments, 50 μL of a suspension of 10^5^ cells/mL were washed in PBS and resuspended in 500 μL of preheated agarose (37 °C). The agarose/cell suspensions were applied to microscopic slides and left for 30 min at 4 °C to allow gel polymerization. After cell lysis using a commercial buffer (Trevigen, Gaithersburg, USA), single-cell gel electrophoresis was performed at 1 V/cm for 1 h. After precipitation of the DNA with ammonium acetate, samples were fixed in 70% ethanol for 30 min and dried. To visualize dsDNA, SYBR™ Green I Nucleic Acid Gel Stain (Invitrogen, Carlsbad, USA) was used. Pictures of at least 50 cells/section were taken using a fluorescence microscope (DMRX, Leica: 450–490 nm BP- filter, 40 × magnification). The extent of the genotoxic effect of PCBs was calculated using the ImageJ-based OpenComet software.

### Micronucleus assay

Transgenic HEK293CYP2A6 and HEK293CYP2C9 cells were cultured in T25 flasks, with a seeding density of 1 × 10^6^ cells per flask at 37 °C with 5% CO_2_. Cells were allowed to grow for 24 h geneticin free before PCBs were added (20 µM or 30 µM). Controls included cells grown in the presence of 1% ethanol (vol/vol) (negative control) or 1% 4-nitroquinoline 1-oxide (positive control). Following further incubation for 24 h, cells were synchronized by adding Cytochalasin B. Subsequently, cells were harvested by trypsinization, centrifuged (2,000*g*, 10 min), and washed with DPBS (Dulbecco’s PBS). For hypotonic shock, 4 °C cold potassium chloride solution was added to the cells under agitation. Cells were completely resuspended and incubated at room temperature for 5 min. After centrifugation (2,500*g*, 10 min), the supernatant was discarded. To fix the cells, they were treated with a methanol/acetic acid solution (− 20 °C), with a minimum incubation time of 15 min at room temperature. Following centrifugation (2,500*g*, 10 min), the supernatant was discarded, and the fixation process was repeated at least three times. Finally, the samples were dissolved in 1 mL of fixative solution and incubated overnight at 4 °C. For analysis, two to three drops from each sample were placed on a glass slide, with intermittent heating of the slide. Subsequently, the cells were visualized with acridine orange, and using a fluorescence microscope (Leica DM6000B, Filter I3 450–490 nm), 2,000 cells per sample were counted.

### Clinical study design

Ten subjects with prior occupational exposure to PCBs and ten controls were included into a clinical CYP phenotyping cocktail study at the Institute for Occupational, Social and Environmental Medicine, University Hospital RWTH Aachen. PCB-exposed participants were reinvited from the HELPcB (Health Effects in High-Level Exposure to PCB) medical surveillance program. Initiated in 2010 by a German Statutory Accident Insurance and a district council, the program responded to human biomonitoring findings that revealed increased PCB blood levels in employees of a capacitor and transformer recycling company, their relatives, and workers from nearby companies (Kraus et al. 2012; Rengelshausen et al. 2023). Participants were requested to enter the study fasted, where they received a single oral administration of a drug cocktail containing CYP phenotyping compounds to evaluate the individual activities of CYP1A2/CYP2A6 (50 mg caffeine), CYP3A4 (1 mg midazolam), CYP2C9 (2.5 mg torasemide), CYP2C19 (10 mg omeprazole), CYP2B6 (50 mg efavirenz), and CYP2D6 (12.5 mg metoprolol). Subjects were allowed to consume non-caffeinated beverages and received a meal 3 following the administration of the drug cocktail. Concentrations of the test compounds and their CYP-mediated metabolites were measured using LC–MS, with urine sampling over 24 h with 5 time points at 1,2,4,6, and 24 h after drug cocktail intake. All participants provided informed consent. Urine samples were weighed during sampling. The study received approval from the ethical committee of the RWTH Aachen (EK 066/22) and is documented in the German Clinical Trials Register with the registration number DRKS00028922, where a description of the study can be found. A manuscript including a detailed description of the study design is in preparation.

### Determination of 1,7-dimethyluric acid/paraxanthine ratios in urine samples

20 µL urine or calibrators and quality controls in Sigmatrix urine diluent (SAE0074-1L, Sigma-Aldrich) were protein precipitated with 80 µL methanol including 1000 ng/mL internal standards caffeine-d9, paraxanthine-d6, and 1,7-dimethyluric acid-d3. Samples were briefly vortexed and centrifuged for 5 min at 17,000*g* and 4 °C. Afterward, 50 µL of the supernatant was diluted in 450 µL 0.1% formic acid in water and again briefly vortexed and centrifuged for 5 min at 17,000*g*, and 4 °C. 100 µL of the supernatant were transferred to an LC vial and 5 µL of the sample were injected for analysis. Urine samples were analyzed with UHPLC-ESI–MS/MS (Agilent 1290 Infinity II UHPLC coupled to a SCIEX QTRAP6500 + triple quadrupole mass spectrometer). Samples were separated with an Accucore C18 column (3 × 100 mm, 2.6 µm particle size; TF17126-103030, Thermofisher Scientific) using the mobile phases A: 0.1% formic acid in water and B: methanol. The flow rate was 0.6 mL/min with a total run time of 7 min per sample. The gradient was as follows: 0 min 5% B with gradual increase to 18% B at 3.9 min, gradual increase to 60% B at 4.1 min, followed by gradual increase to 95% B at 4.5 min, 95% B was kept till 5.5 min and then re-equilibration to 5% B at 5.6 min, 5% B was kept till 7 min. The first 2 min of each sample run were diverted to waste and acquisition was stopped after 5.5 min. Analytes were quantified using scheduled multiple reaction monitoring (sMRM). Caffeine and paraxanthine were detected in positive ion mode and 1,7-dimethyluric acid in negative ion mode. MRM transitions for caffeine were 195 → 138 (quantifier), 195 → 110 (qualifier), and 204 → 144 (internal standard). MRM transitions for paraxanthine were 181 → 124 (quantifier), 181 → 96 (qualifier), and 187 → 127 (internal standard). MRM transitions for 1,7-dimethyluric acid were 195 → 180 (quantifier), 195 → 137 (qualifier), and 198 → 183 (internal standard). The method was validated for intra- and inter-day accuracy and precision, stability, matrix effect, and dilution integrity (more detailed information in supplemental data). Validation data for 1,7-dimethyluric acid and paraxanthine are shown in Fig. S3 and Tables S2–S5. The metabolic ratio for 1,7-dimethyluric acid/paraxanthine was calculated based on the measured urine concentrations. The total excreted amount between collection time points (0–360 min) was summed, and the data underwent logarithmic transformation.

### Pharmacogenetic analysis

Genotyping was performed at the Dr. Margarete Fischer-Bosch-Institute for Clinical Pharmacology, Stuttgart. CYP2A6 genotypes were extracted from the Infinium™ Global Screening Arrays (GSA)-v2.0 (Illumina Inc., San Diego, USA) using pypgx 0.19.0 and the chip-pipeline with the impute option and 1000 Genomes Project (1KGP) data as reference covering the most important alleles (i.e., *2, *9, *12, *17) (21). In addition, using the TaqMan copy number assay Hs07545274_cn within intron 1, CYP2A6 deletions (*4, *12) and gene duplications were determined.

### Statistical analysis

Statistical analysis was performed by an unpaired, two-tailed Student’s *t* test for MTT assay and micronucleus formation. *T* tests were corrected for multiple comparisons using the Benjamini, Krieger, and Yekutieli (BKY) method to control the false discovery rate (FDR). For comet assay, differences of the medians between samples and negative control were investigated using Kruskal–Wallis test, followed by a Dunn’s post hoc test for multiple comparisons. All statistical tests performed were designated in the respective figure legends.

## Results and discussion

### Metabolic profiling of PCBs in engineered HEK293 cell lines

Considering the isoform-specific and atroposelective biotransformation of various PCB congeners by CYPs, we aimed to identify the isoforms catalyzing the metabolization of WHO PCB congeners PCB28, PCB52, and PCB101 in vitro. We, therefore, modified HEK293 cells through genetic engineering to express CYP1A2, CYP2C8, CYP2C9, CYP3A4, CYP2A6, and CYP2E1. We then demonstrated the functionality of the transgenic cell lines by the successful conversion of well-established specific substrates for the respective CYPs (Table 1). The CYP overexpressing cell lines metabolized the probe drug substrates as demonstrated by formation of the specific metabolites (Table 1 and Fig. S1).

**Table 1:** Functional assessment of CYP-isoenzyme transgenic HEK293 cells metabolizing known CYP isoenzyme-specific substrates

Cell line	Substrate	Retention time	Conversion product	Retention time
HEK293CYP1A2	Caffeine	2.2	Paraxanthine	1.6
HEK293CYP2C8	Amodiaquine	2.3	Desethylamodiaquine	2.1
HEK293CYP2C9	Torasemide	7.0	Hydroxy torasemide	3.1
HEK293CYP3A4	Midazolam	6.4	1-hydroxy-midazolam	6.7
HEK293CYP2A6	Coumarin	3.5	7-hydroxycoumarin	2.9
HEK293CYP2E1	Chlorzoxazone	5.5	6-hydroxychloroxazone	2.2

**Fig. 1:**
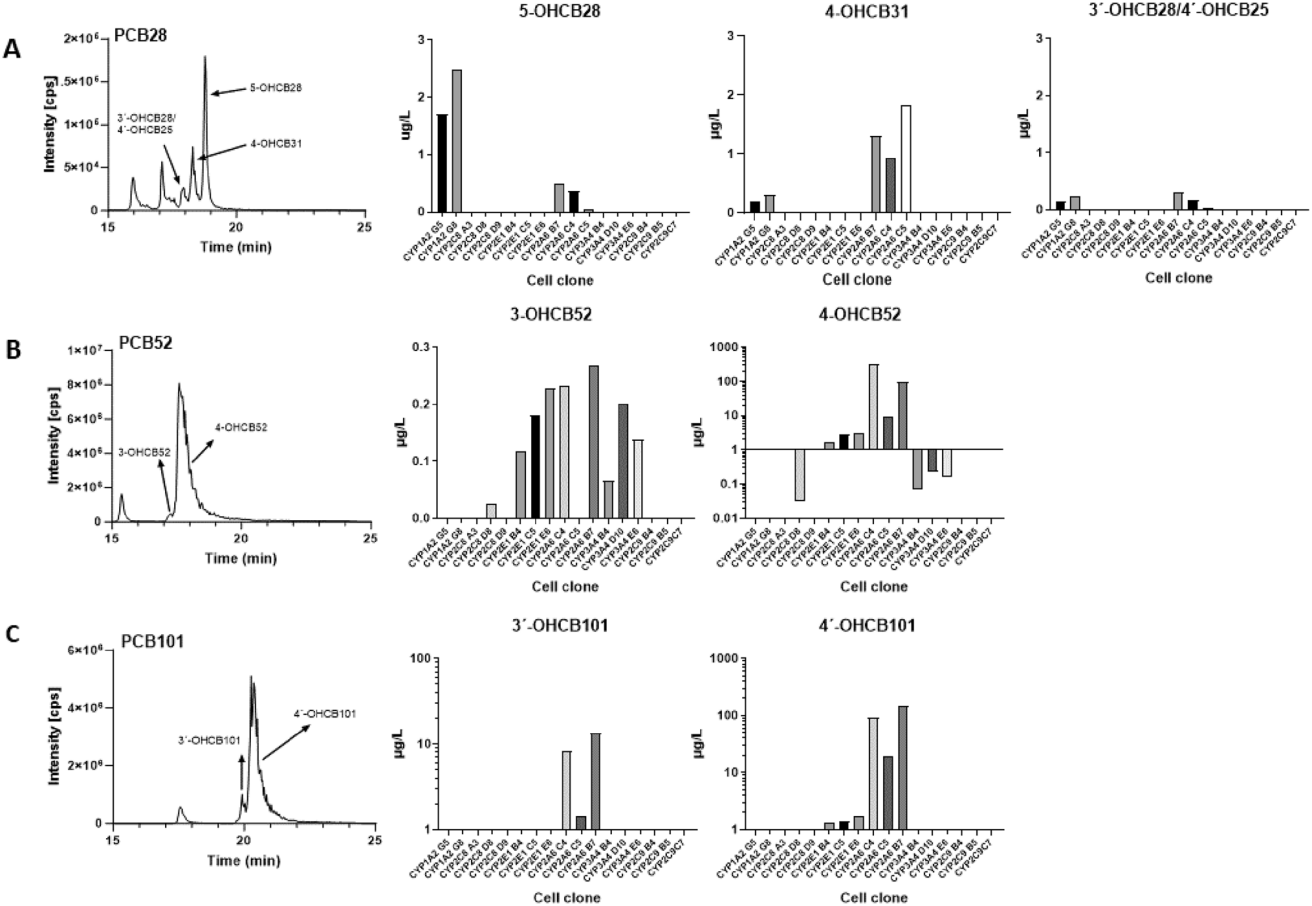
Metabolism of PCB28, PCB52, and PCB101 in transgenic HEK293 cells stably expressing human CYP*. Single-cell*-derived *clonal* lines harboring CYP1A2, CYP2C8, CYP2E1, CYP2A6, CYP3A4, and CYP2C9 were incubated with 20 µm of either PCB28, PCB52 or PCB101 for 24 h with subsequent collection of the supernatant. **A** Production of the PCB28-derived metabolites 5-OHCB28, 4-OHCB31, 3′-OHCB28 4′-OHCB25 across various cell clones, **B** production of the PCB52-derived metabolites 3-OHCB52 and 4-OHCB52, and **C** production of the PCB101-derived metabolites 3′-OHCB101 and 4′-OHCB101. Representative LC/MS chromatograms of OH-PCB-metabolites are shown

Once functionality of the generated cell lines was confirmed, we subjected them to a 24-h exposure to the different PCB congeners in question (Fig. 1 and Table S1). To eliminate potential phenotypic variations in PCB metabolism within transgenic cell lines, we assessed at least two independently established cell clones for each CYP, all established through limiting dilution. Regarding PCB28, our findings revealed that HEK293CYP1A2 cell clones are primarily responsible for the formation of the main human metabolite 2,4,4′-trichloro-5-biphenylol (5-OHCB28; RT 19.14; [1.7–2.48 µg/L], [6.22–9.07 nM]) and only to a lesser extent for the metabolism to the NIH 1,2-shift products 2,4′,5-trichloro-4-biphenylol (4-OHCB31; RT 18.66; [0.19–0.30 µg/L], [0.69–1.11 nM]) or 2′,3′,4-trichloro-4′-biphenylol (4′-OHCB25; RT 17.93; in sum [0.15–0.23 µg/L], [0.56–0.84 nM]) (coeluting with 2,4,4′-trichloro-3′-biphenylol, 3′-OHCB28 RT 17.93) (Fig. 1 and Table S1). Similarly, we produced all four metabolites from PCB28 in our previous experiments when we used bacterially expressed CYP1A2 as a monooxygenase (Idda et al. 2020). On the other hand, HEK293CYP2A6 cell lines were producing the 1,2-shift metabolites of PCB28 at considerably higher rates compared to HEK293CYP1A2 cells (4-OHCB31; [0.18–1.31 μg/L], [0.66–4.8 nM] and 4′-OHCB25/3′-OHCB28; [0.03–0.3 μg/L], [0.13–1.1 nM]) (Fig. 1 and Table S1). This is in line with observations of Uwimana and colleagues reporting CYP2A6 as mainly responsible for metabolism of PCB91 to the 1,2-shift product 3-OHCB100 (Uwimana et al. 2019)**.** In addition, both HEK293CYP1A2 and HEK293CYP2A6 cell clones formed 4,4′-dichlorobiphenyl (3-OHCB15), resulting from partial dechlorination of PCB28 (data not shown; Randerath et al. 2024).

The metabolic processing of PCB52 and PCB101 was carried out by cell lines expressing CYP3A4, CYP2E1, and CYP2A6 with the order CYP2A6 > CYP2E1 > CYP3A4. The HEK293CYP2C8 cell line exhibited very limited metabolic activity toward both congeners with mostly producing metabolite levels below the limit of quantification. In contrast, HEK293CYP2A6 cells demonstrated a notably robust metabolism of all three PCB congeners examined in this study. Both for PCB52 and PCB101, hydroxylation at the para-position 4-hydroxy-2,2′,5,5′-tetrachlorobiphenyl (4-OHCB52; RT 17.98; [9.48–316.89 µg/L], [10.23–1029.6 nM]) and 4′-hydroxy-2,2′,4′,5,5′-pentachlorobiphenyl (4′-OHCB101; RT 20.77; [19.15- 151.1 µg/L], [55.93–441.3 nM]) was by far the main metabolite formed with levels up to 3 orders of magnitude higher compared to the meta-position 3-hydroxy-2,2′,5′5′-tetrachlorobiphenyl (3-OHCB52; RT 17.12; [0.23–0.27 µg/L], [0.74–0.87 nM]) and 3′-hydroxy-2,2′,4′,5,5′-pentachlorobiphenyl (3′-OHCB101; RT 20.2; [1.44–13.5 µg/L], [4.2–39.4 nM]) (Fig. 1 and Table S1). Interestingly, our own investigations on individuals with high occupational exposure to PCBs (Schettgen et al. 2012b) revealed that 4-OHCB52 was the only metabolite detectable in human plasma, while both metabolites (3-OHCB52 and 4-OHCB52) were detectable in human urine, pointing to a more efficient retention of the 4-OH-metabolites in blood under physiological conditions as reported before (Quinete et al. 2017). Metabolization of PCB101 and PCB52 in our studies is in line with previous results using bacterially expressed recombinant CYP2A6. Shimada et al. investigated the role of CYP2A6 in the oxidation of PCB52, contrasting its activity with other CYPs (Shimada et al. 2016). Consistent with our own findings, which demonstrated increased metabolization rates by CYP2A6 in comparison to CYP2E1 and CYP3A4, Shimada et al. observed a significantly elevated oxidation rate of PCB52 by CYP2A6 relative to other CYPs, notably CYP2A13. Furthermore, McGraw and Waller identified CYP2A6 as the enzyme responsible for oxidizing PCB101 to form the para-metabolite 4′-OHCB101, with no detection of the meta-position 3′-OHCB101 (McGraw and Waller 2006). Moreover, studies have indicated that CYP2A6 facilitates the oxidation of PCB91 and PCB132 in the meta-position, and PCB95 and PCB136 in the para-position (Uwimana et al. 2019). Altogether, the results of these studies, in conjunction with our own findings, reinforce the concept that CYP2A6 plays a pivotal role in the metabolism of PCB congeners, without displaying structural preferences. However, CYP2A6 seems to favor catalyzing the aromatic ring hydroxylation of arene oxide metabolites (NIH-shift products), predominantly leading to the selective formation of para-position OH-metabolites.

### PCB metabolism induced toxicity in HEK293CYP2A6 cells

**Fig. 2:**
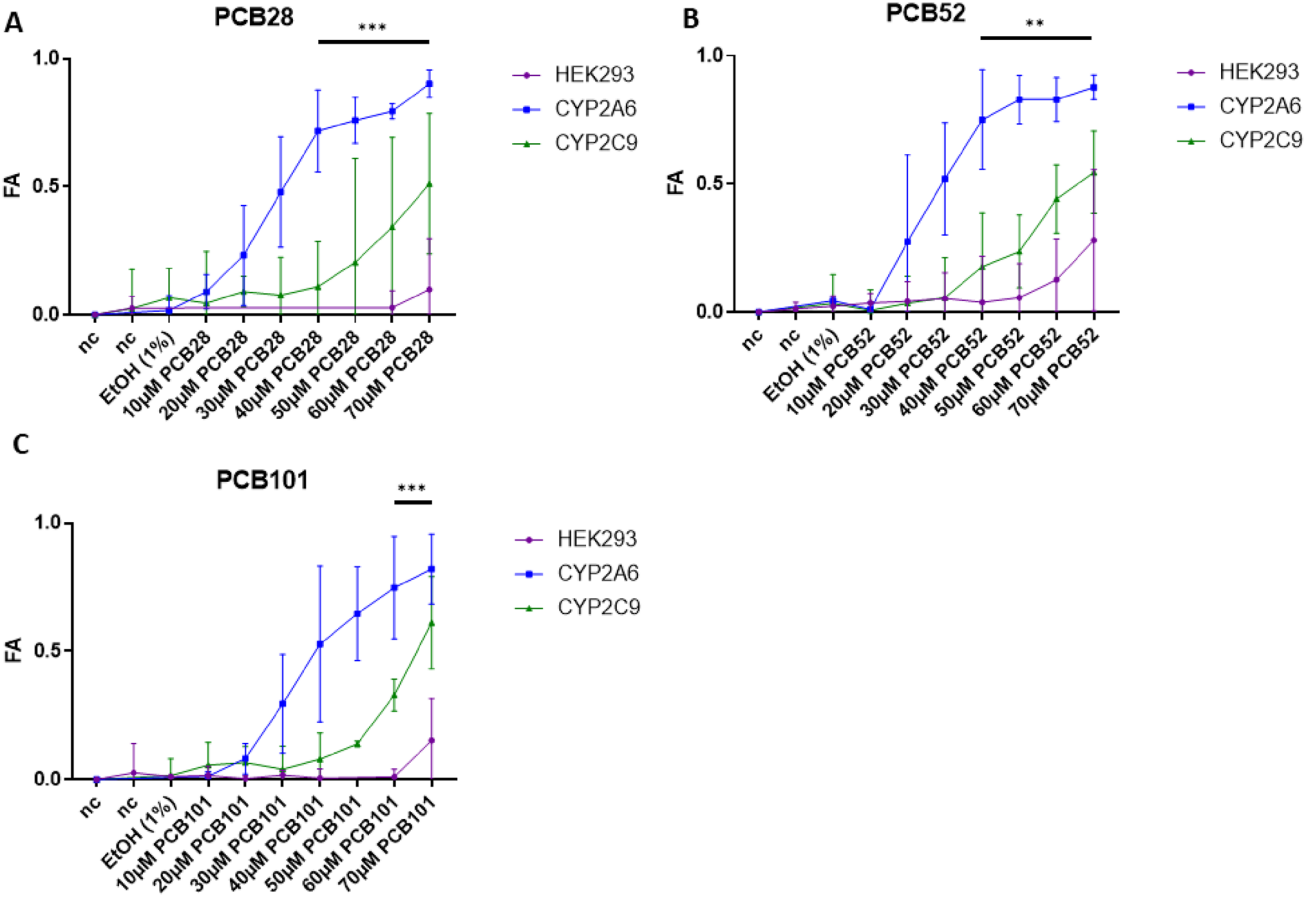
Inhibition of metabolic activity in HEK293CYP2A6 cells by activation of PCB28, PCB52, and PCB101. HEK293 WT cells, transgenic HEK293CYP2A6 cells, and transgenic HEK293CYP2C9 cells were incubated with increasing concentrations of PCB28 (**A**), PCB52 (**B**), and PCB101 (**C**). Mitochondrial and cellular viability were measured by MTT assay. Mean ± SD of five different experiments each with a total of 6 replicates are shown. Statistical analysis was performed by an unpaired, two-tailed Student’s *t* test. The *t* tests were corrected for multiple comparisons using the Benjamini, Krieger, and Yekutieli (BKY) method to control the false discovery rate (FDR), and significant differences between HEK293 cells and CYP2A6 transgenic cells were observed, becoming statistically significant at a PCB concentration of 40 µM (PCB28, PCB52) and a concentration of 60 µM (PCB101) respectively. Statistically significant differences are indicated (**P < 0.01; ***P < 0.001)

Following the comprehensive metabolic profiling of PCBs in engineered HEK293 cell lines, we proceeded to investigate the biological consequences of these metabolic processes. Specifically, we aimed to understand the impact of PCB hydroxylation by CYP2A6 on cellular viability and metabolic activity. By conducting a series of toxicity assays, we compared the effects observed in HEK293CYP2A6 cells with those in control cell lines, including HEK293CYP2C9 and HEK293 cells. This approach allowed us to elucidate the cytotoxic and cytostatic effects of the hydroxylated metabolites of PCB28, PCB52, and PCB101, highlighting the significant role of CYP2A6 in mediating these toxicological responses. Initially, we employed MTT assays to investigate the potential influence of endogenous PCB biotransformation on cellular viability or metabolic activity after 24 h of incubation (Fig. 2). Within HEK293CYP2A6 cells, the fraction of cell viability affected (FA) increased for all three PCB congeners tested, starting at a concentration of 10 µM PCB28 (Fig. 2A) and 20 µM for both PCB52 (Fig. 2B) and PCB101 (Fig. 2C). FA in HEK293CYP2A6 cells exhibited a marked increase for all PCBs between concentrations of 20 and 40 μM (FA ≥ 60%), followed by a gradual transition into a plateau phase. The IC_50_ values for PCB28, 52, and 101 in the HEK293CYP2A6 cell line were determined to be 26.98 μM, 26.15 μM, and 34.82 μM, respectively. Conversely, noticeable effects on cell viability or metabolic activity (FA ≥ 10%) were noted in the control cell line HEK293CYP2C9 for all congeners tested starting at a concentration of 40 μM (Fig. 2A–C). Despite increasing PCB concentrations, FA levels in HEK293CYP2C9 cells remained consistently lower than those in HEK293CYP2A6 cells, even at the highest concentrations of PCBs tested. Effects on metabolic activity in non-transgenic HEK293 cells were observed only at exceptionally high concentrations of PCBs (Fig. 2A–C). These results show that hydroxylation markedly alters how the WHO-congeners PCB 28, 52, and 101 influence cellular viability or metabolic activity. Considering that HEK293CYP2A6 cells yields roughly 1.2 μg/L (4.4 nM) of hydroxylated metabolites from 20 μM of PCB28 overall (Table S1), compared to approximately 141.8 μg/L (460.4 nM) and 95.25 μg/L (143.8 nM) from 20 μM of PCB52 and PCB101, respectively, it becomes apparent that PCB28 metabolites possess the highest toxic potency among the three WHO-congeners. In addition, we performed a light microscopic evaluation of HEK293CYP2A6 cells after 24 h of incubation with PCBs which revealed primarily cytotoxic effects (cell death) for PCB28 and cytostatic effects (proliferation arrest) for PCB52 and 101 (data not shown).

**Fig. 3:**
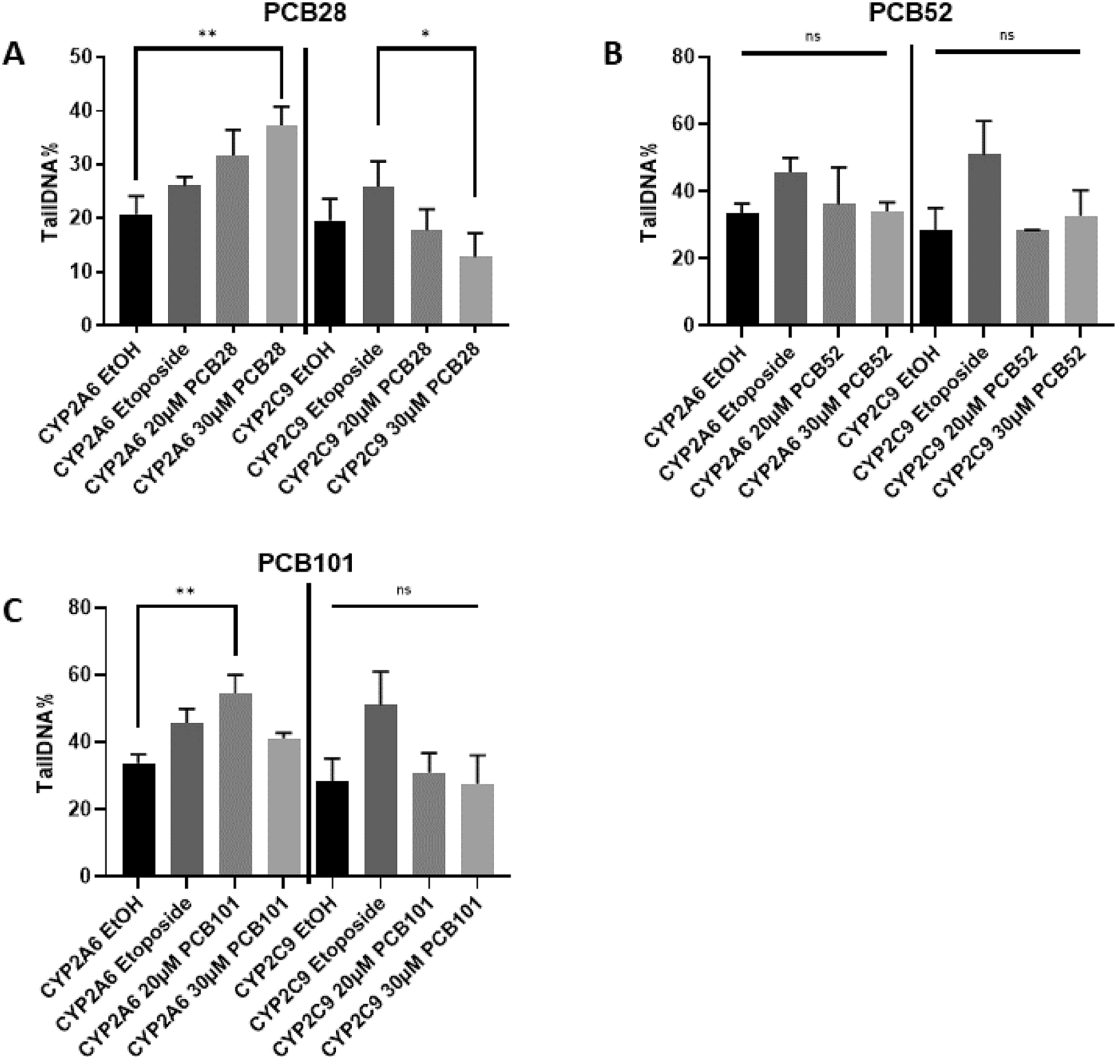
Induction of DNA damage in HEK293CYP2A6 cells by activation of PCB28 and PCB101. Transgenic HEK293CYP2A6 cells and transgenic HEK293CYP2C9 cells were either incubated with 20 or 30 µm PCB28, PCB52, and PCB101 or 10 µm etoposide as a positive control. After 5 h of incubation, a comet assay was performed. Percentage of tail DNA is represented in histograms. Mean ± SD of three different experiments are shown. Differences of the medians in comparison to negative control were investigated using Kruskal–Wallis test, followed by a Dunn’s post hoc test for multiple comparisons. Statistical differences between certain treatment groups are indicated (*P < 0.05, **P < 0.01). NS, no significance between the four treatment groups

Understanding the cytotoxic and cytostatic effects of PCB metabolites provided a foundation for further investigating the genetic stability implications of these metabolic processes as genotoxicity is induced by reactive intermediates of the PCB hydroxylation pathway like quinones, glucuronides, and methoxylated metabolites (Shimada et al. 1981; Safe 1994; Srinivasan et al. 2001). Quinones can directly interact with DNA, forming adducts, while glucuronides and methoxylated metabolites can increase oxidative stress, leading to DNA damage (Ludewig et al. 2008). Consequently, we expanded our research to assess the genotoxic potential of the PCB hydroxylation pathway using comet and micronucleus assays for DNA damage (Fig. 3 and supplemental Fig. 2A). When HEK293CYP2A6 cells were exposed to respective PCB congeners at concentrations of 20 or 30 µM for 3 h, subsequent comet assays demonstrated a concentration-dependent increase in the percentage of tail DNA for PCB28 above background levels which was not evident in HEK293CYP2C9 control cells (Fig. 3A and supplemental Fig. 2A). DNA damage was also seen in HEK293CYP2A6 cells exposed to PCB101 (Fig. 3B) but not for PCB52 (Fig. 3C). Using the percentage of tail DNA as a reference point, we classified resulting DNA damage according to the comet scale as follows: PCB28 at 20 µM and 30 µM exhibited moderate damage, PCB52 at 20 µM and 30 µM showed low damage, and PCB101 at 20 µM displayed high damage, while 30 µM showed moderate damage. Notably, the genotoxicity of PCB28 and 101 in HEK293CYP2A6 cells was more potent than of etoposide (an inhibitor of topoisomerase II and a very powerful staple of chemotherapy) (Fig. 3A, C). Based on our prior experiments employing chemically synthesized 5-OHCB28 and 3′-OHCB28 to induce DNA double-strand breaks (DSB) in Jurkat T cells and proliferating lymphocytes (as reported by Vasko et al. 2018), the above results indicate that among a series of CYPs important for the metabolism of xenobiotics, CYP2A6 (naturally expressed in human hepatocytes) is specifically capable of activating PCBs to genotoxic metabolites.

**Fig. 4:**
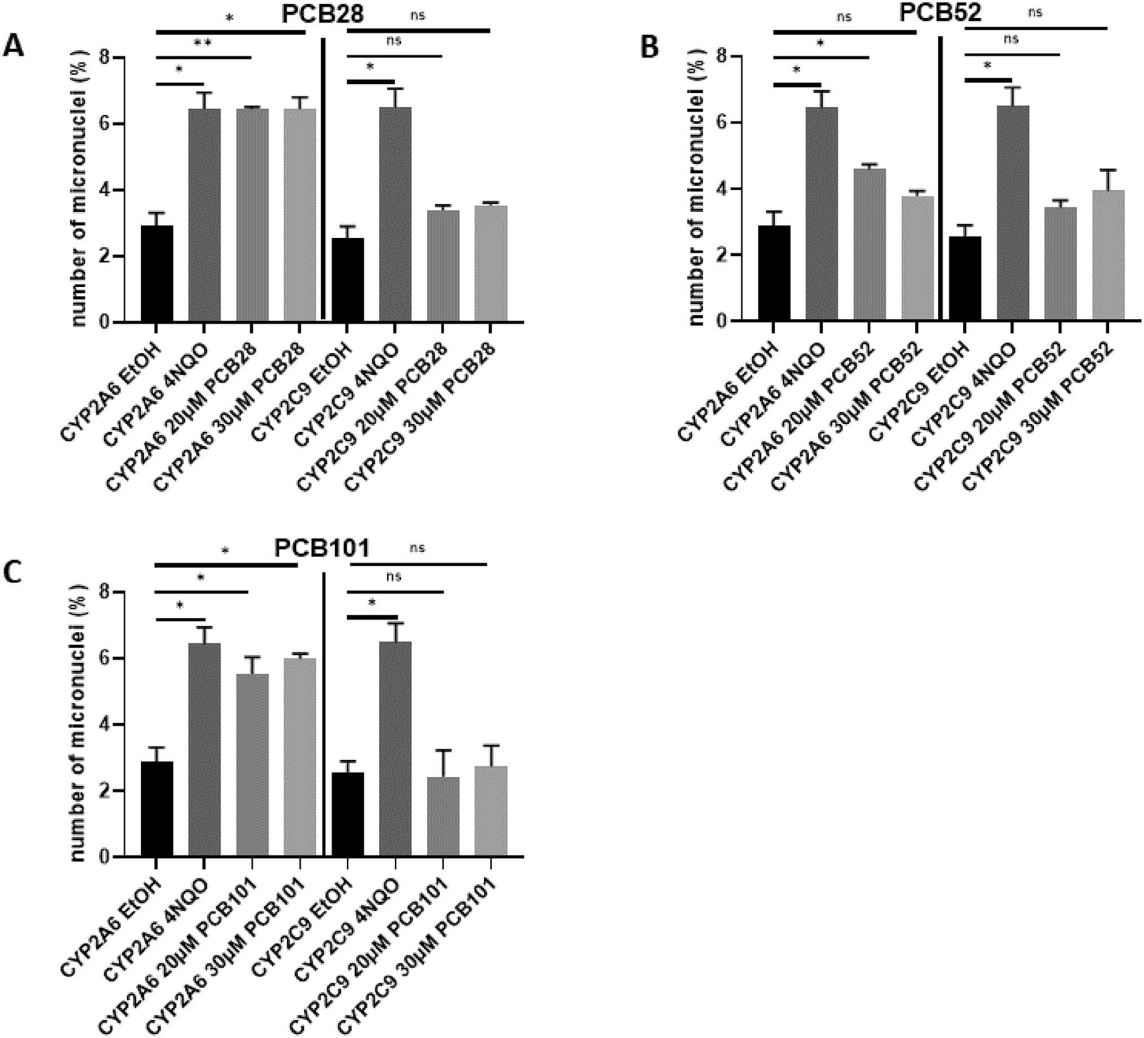
Frequency of micronucleus formation in PCB-treated HEK293CYP2A6 cells. Transgenic HEK293CYP2A6 cells and transgenic HEK293CYP2C9 were incubated with 20 or 30 µm PCB28, PCB52, and PCB101 or 10 µm 4-nitroquinoline as a positive control for 24 h. Subsequently cytokinesis was blocked by adding Cytochalasin B for 24 h. Percentage of cells with micronucleus per 2000 counted cells is shown. Mean ± SD of three different experiments are shown. Statistical analysis was performed by an unpaired, two-tailed Student’s *t* test. Statistically significant differences are indicated (**P < 0.01; ****P < 0.0001)

To assess whether genetic instability caused by PCB metabolism is retained during mitosis and transmitted to daughter cells, we performed micronucleus assays. PCB28 and PCB101 induced approximately threefold elevated percentages of micronuclei in HEK293CYP2A6 cells at 20 and 30 µM (Fig. 4A, B and supplemental Fig. 2B), while being inactive in HEK293CYP2C9 cells. PCB52 (Fig. 4C) exhibited a diminished effect in HEK293CYP2A6 cells, noticeable only at a concentration of 20 µM but with indistinguishable effects in HEK293CYP2C9 control cells.

The control experiments underscored a consistent and significant elevation of the positive control 4-nitroquinoline-(1)-oxide (a quinoline derivative with potent tumorigenic activity) when compared to the negative control ethanol (Fig. 4A–C). Notably, samples from the control cell line HEK293CYP2C9 stayed within the same range as the negative control, for all PCBs tested (Fig. 4A–C). The JECFA (2016) summarized in vitro genotoxicity studies on WHO PCB congeners PCBs 52 and 101, along with two in vivo genotoxicity studies for PCB52. Out of the in vitro studies, both PCB52 and 101 showed positive results in two studies each, while three studies on PCB52 reported negative results. However, both in vivo studies on PCB52 reported negative genotoxicity findings. For instance, PCB52 and its primary metabolites did not exhibit mutagenic activity in *Salmonella typhimurium* strains (Wyndham et al. 1976). Although PCB52 showed positive or weakly positive results for DNA strand breaks in mouse fibroblasts and human lymphocytes (Stadnicki et al. 1979), it was tested negative for sister chromatid exchange in human lymphocytes (Sargent et al. 1989). In vivo studies with PCB52 also demonstrated the absence of chromosomal aberrations in bone marrow and liver (Sargent et al. 1992). PCB101 was found to induce DNA strand breaks and micronucleus formation in fish cells (Marabini et al. 2011). Furthermore, PCB101 increased significantly genotoxicity in human lung fibroblast at higher concentrations compared to lower concentrations, which was attributed to oxidative stress (Hashmi et al. 2015).

Based on the findings presented and our own analysis, we conclude that PCB28 and PCB101 metabolism initiated by CYP2A6 induces DNA damage likely to propagate genetic defects to subsequent generations, potentially contributing to carcinogenesis.

### In vivo examination and retrospective analysis of CYP2A6 enzyme activity in PCB-exposed individuals

**Fig. 5:**
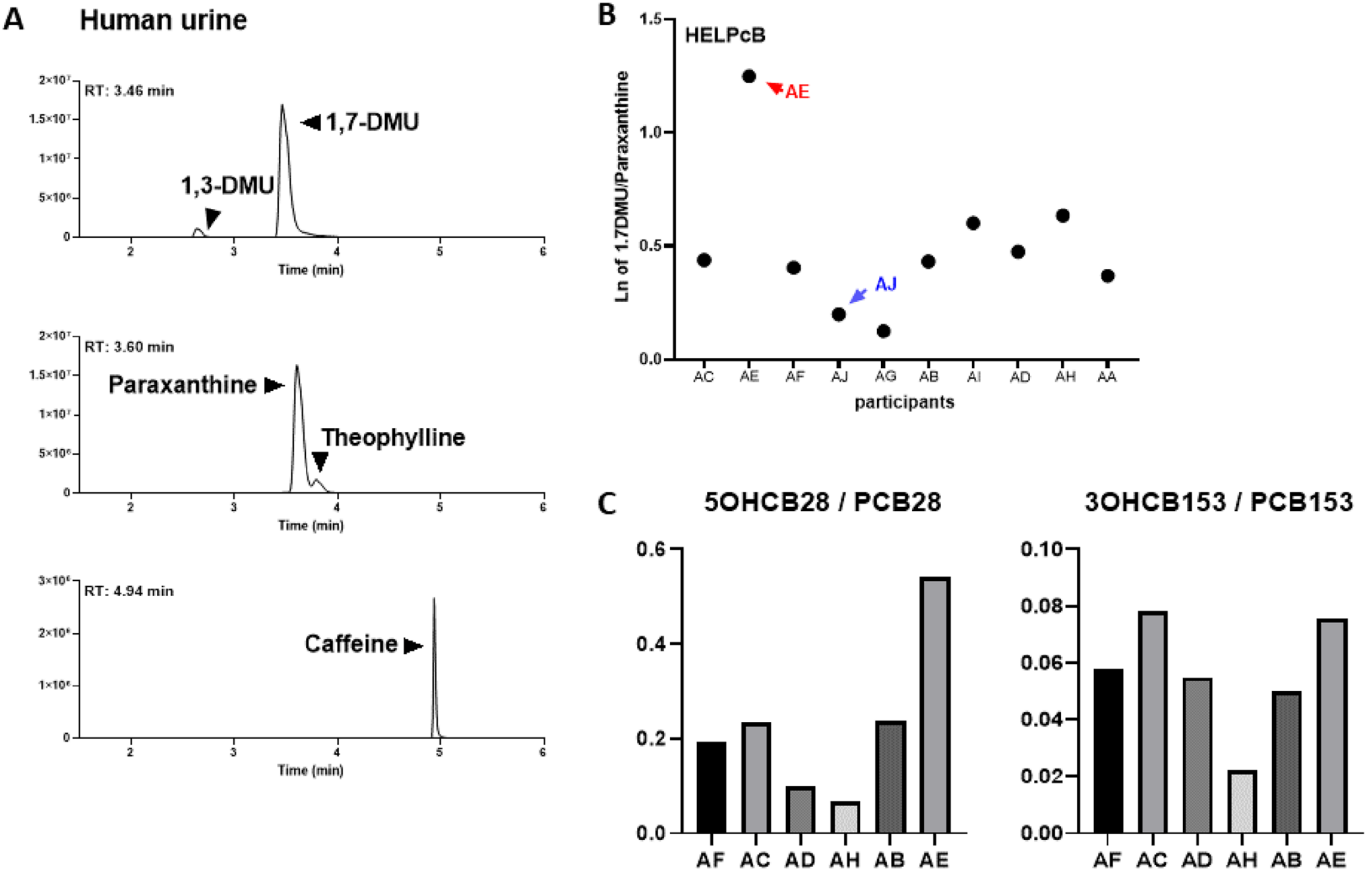
CYP2A6 enzyme activity in PCB-exposed individuals. **A** Representative chromatogram of 1,7-DMU, paraxanthine and caffeine in human urine. Additional signals for 1,3-DMU and theophylline are detected in the MRM transitions for 1,7-DMU and paraxanthine, respectively. RT: retention time, 1,7-DMU: 1,7-dimethyluric acid, 1,3-DMU: 1,3-dimethyluric acid. **B** Ten subjects with prior occupational exposure to PCBs (HELPcB-cohort) were included into a clinical study using caffeine as a probe. CYP2A6 phenotype was estimated by urinary 1,7-dimethyluric acid (1.7DMU)/ paraxanthine ratio. The figure represents a linear depiction of logarithmically transformed data (Ln). **C** Retrospective analysis of PCB28 and PCB153 metabolism in samples from participants of the clinical study taken in 2010. Metabolic ratios of 5-OH-PCB28/PCB28 and 3-OH-PCB153/PCB153 are shown

With the cytotoxic and genotoxic effects of hydroxylated PCB metabolites established in HEK293CYP2A6 cells, it was important to extend these in vitro findings to an in vivo context to determine their significance for human health. To accomplish this, we studied the activity of CYP2A6 in individuals from the HELPcB-cohort who had been previously exposed to PCBs. By evaluating the metabolic activity of CYP2A6 in participants through both pharmacological and genetic assessments, we sought to correlate the in vitro results with real-world exposure conditions. This thorough approach allowed us to investigate the role of CYP2A6 in PCB metabolism and its potential implications for genetic stability and carcinogenesis in humans. Utilizing caffeine and its metabolites, paraxanthine and 1,7-dimethyluric acid (1,7-DMU), allows for the assessment of metabolic ratios associated with both CYP1A2 and CYP2A6 enzyme activity (Begas et al. 2007). 1,7-DMU/paraxanthine metabolic ratio in urine samples is specifically employed to evaluate CYP2A6 (Nowell et al. 2002). Building upon the established role of CYP2A6 in vitro metabolism of WHO PCB congeners PCB28, PCB52, and PCB101, we took advantage of caffeine intake within a clinical study to probe the in vivo activity of CYP2A6 in subjects with former exposure to commercial PCB mixtures (HELPcB-cohort) (Kraus et al. 2012; Rengelshausen et al. 2023). Pharmacological determination of enzyme activity was conducted on ten individuals following a single dose of caffeine administration. Simultaneously, genotyping analysis based on known CYP2A6 genotypes was performed to explore potential impacts on paraxanthine metabolism. Analysis of urine samples resulted in the successful detection of CYP2A6 and CYP1A2 specific metabolites 1,7-DMU and paraxanthine (Fig. 5A). Logarithmically transformed metabolic ratios of 1,7-DMU and paraxanthine across participants were found to be between 0.3 (presumably low CYP2A6 activity) and 1.3 (presumably very high CYP2A6 enzyme activity) with a median metabolic ratio of (Ariyoshi et al. 1995) 0.4 (Fig. 5B).

Predicted phenotypes based on identified alleles are presented in Table 2, with additional identification of all genotypes diverging from the wild type. Participant AJ (highlighted in blue) exhibited only one functional allele in the *1/*12 genotype, which was expected to lead to a decreased enzyme activity in vivo and indeed resulted in a low 1,7-DMU/paraxanthine metabolic ratio as shown in Fig. 5B. In contrast, participant AE (highlighted in red) demonstrated a marked rise in CYP2A6 enzyme activity, which could not be attributed to any specific genotype. To determine if the 1,7-DMU/paraxanthine metabolic ratio measured in the participants of this study reflects CYP2A6 enzyme activity associated with the metabolism of PCB-congeners PCB28, 52, or 101 in vitro, we analyzed plasma samples from participants of the study dating back to 2010, representing the peak PCB exposure in the HELPcB-cohort. Analysis of samples revealed elevated PCB28 levels in six out of ten study participants with no detection of PCB52 or 101, prompting a thorough investigation into PCB28 metabolism in this subset (supplementary Table 1). To increase the specificity of the analysis in respect to CYP2A6 enzyme activity, we also examined the metabolism of PCB153, a PCB congener that, based on current knowledge, is not a substrate of CYP2A6. Thus, we examined the metabolic ratio of 5-OHCB28/PCB28 and, as a control, 3-OHCB153/PCB153 within this group (Fig. 5C). By analysis of the 5-OHCB28/PCB28 metabolic ratio, it can be demonstrated that study participant AE exhibits significantly elevated specific metabolic activity in relation to the PCB28 substrate, which in contrast was not observed in the metabolism of PCB153 among the study cohort (Fig. 5C). We conclude that there is a correlation between real-time paraxanthine metabolism and retrospective PCB28 metabolism in study participant AE and attribute it to elevated CYP2A6 enzyme activity. Based on this result and the in vitro findings demonstrated earlier, we, therefore, suggest that CYP2A6 serves as a critical enzyme for the in vivo metabolism of low-chlorinated PCB congeners in humans.

**Table 2:** Analysis of CYP2A6 genotype and predicted phenotype among study participants (phenotype prediction was done according to Tanner and Tyndale (2017) and (Zanger and Schwab (2013)

ID	CN calculated	CN predicted	Diplotype	Genotype predicted phenotype
HELPcB				
AA	2.06	2	*1/*1	Normal
AB	1.94	2	*1/*1	Normal
*AC*	*1.92*	*2*	*1/*9	Decreased/normal
AD	2.08	2	*1/*1	Normal
AE	1.98	2	*1/*1	Normal
AF	1.94	2	*1/*1	Normal
AG	1.93	2	*1/*1	Normal
AH	1.99	2	*1/*1	Normal
AI	2.03	2	*1/*1	Normal
*AJ*	*0.93*	*1*	*1/*12	Decreased

Italic values indicate genotypes that differ from the wildtype

ID, identification number; CN, copy number

Although we could not identify a specific genotype responsible for the increased CYP2A6 enzyme activity, this further suggests that there are individual differences in PCB metabolism. Several epidemiological studies have explored the intricate link between genetic variability and the carcinogenic effects of PCBs, particularly focusing on polymorphisms in genes like CYP1A1 and their association with breast cancer risk following PCB exposure (Moysich et al. 1999; Laden et al. 2002; Zhang et al. 2004). For example, one study examined the correlation between PCB exposure, CYP1A1 polymorphism, and post-menopausal breast cancer risk. Although no independent association between the CYP1A1 genotype and breast cancer risk was found, a significant increase in risk was observed with PCB exposure, particularly in women with higher PCB serum concentrations and at least one valine allele compared to those homozygous for isoleucine alleles. These findings suggest that interactions between genetic variations and PCB exposure may influence breast cancer risk (Moysich et al. 1999).

For CYP2A6, genetic variations in the population are known which may affect the efficacy and toxicity profiles of CYP2A6 substrates (Tanner and Tyndale 2017). In PCB28-exposed individuals with increased activity of CYP2A6, an elevated formation of activated metabolites, such as 3-OHCB15 (Randerath et al. 2024), which has demonstrated tumorigenic properties in animal studies (Espandiari et al. 2003), and thus an increased adverse effect of PCB28 is likely to be expected. However, the significance of specific known genetic variations in these enzymes for assessing individual disease risks following PCB exposure is not yet clear and should be investigated in further studies.

## Conclusion

Our study identified CYP2A6 as a key enzyme in the biotransformation of PCB28, PCB52, and PCB101. In engineered HEK293 cells, CYP2A6 demonstrated robust activity, hydroxylating all three congeners tested and leading to significant cytotoxic and cytostatic effects. Genotoxicity assays indicated that CYP2A6-mediated metabolism of PCB28 and PCB101 induced notable DNA damage, which was propagated to daughter cells. These findings underscore the pivotal role of CYP2A6 in PCB metabolism and its potential contribution to genetic instability and carcinogenesis. Our results enhance the understanding of how OH-PCBs, through their metabolites, can lead to significant genotoxic effects, emphasizing the need for considering enzyme-specific pathways in risk assessments. In addition to our in vitro experiments, we conducted in vivo studies, albeit with a small sample size, which support our findings and suggest a similar metabolic role of CYP2A6 in humans. While these in vivo results are promising, they also highlight the complexity of metabolic pathways and interactions that may not be fully captured in vitro. Future research should explore the genetic variability of CYP2A6 and other CYP isoforms to better understand individual susceptibilities to PCB-induced health effects. Investigating the in vivo relevance of these findings in larger, more diverse populations and incorporating additional metabolic pathways will further elucidate the health risks posed by PCB exposure.

## Supplementary Information

Below is the link to the electronic supplementary material.

Supplementary file1 (DOCX 346 KB)

## References

[CR1] Anonymous (1999) Stoffmonographie PCB-Referenzwerte für Blut. Bundesgesundheitbl-Gesundheitsforsch-Gesundheitschutz 6:511–521

[CR2] Ariyoshi N, Oguri K, Koga N, Yoshimura H, Funae Y (1995) Metabolism of highly persistent PCB congener, 2,4,5,2′,4′,5′-hexachlorobiphenyl, by human CYP2B6. Biochem Biophys Res Commun 212(2):455–460. 10.1006/bbrc.1995.1991 7626059 10.1006/bbrc.1995.1991

[CR3] Ballschmiter K, Zell M (1980) Analysis of polychlorinated biphenyls (PCB) by glass capillary gas chromatography. Fresenius’ Z Anal Chem 302:20–31

[CR4] Beck H, Mathar W (1985) Analysenverfahren zur Bestimmung von ausgewählten PCB-Einzelkomponenten in Lebensmitteln. Bundesgesundheitsblatt 28:1–12

[CR5] Begas E, Kouvaras E, Tsakalof A, Papakosta S, Asprodini EK (2007) In vivo evaluation of CYP1A2, CYP2A6, NAT-2 and xanthine oxidase activities in a Greek population sample by the RP-HPLC monitoring of caffeine metabolic ratios. Biomed Chromatogr 21(2):190–200. 10.1002/bmc.736 17221922 10.1002/bmc.736

[CR6] Carpenter DO (2006) Polychlorinated biphenyls (PCBs): routes of exposure and effects on human health. Rev Environ Health 21(1):1–23. 10.1515/reveh.2006.21.1.1 16700427 10.1515/reveh.2006.21.1.1

[CR7] EFSA (2005) Opinion of the Scientific Panel on contaminants in the food chain related to the presence of non dioxin-like polychlorinated biphenyls (PCB) in feed and food. Eur Food Saf Auth J 3:284

[CR8] Espandiari P, Glauert HP, Lehmler HJ, Lee EY, Srinivasan C, Robertson LW (2003) Polychlorinated biphenyls as initiators in liver carcinogenesis: resistant hepatocyte model. Toxicol Appl Pharmacol 186(1):55–62. 10.1016/s0041-008x(02)00018-2 12583993 10.1016/s0041-008x(02)00018-2

[CR9] Esser A, Ziegler P, Kaifie A, Kraus T, Schettgen T (2021a) Estimating plasma half-lives of dioxin like and non-dioxin like polychlorinated biphenyls after occupational exposure in the German HELPcB cohort. Int J Hyg Environ Health 232:113667. 10.1016/j.ijheh.2020.113667 33307299 10.1016/j.ijheh.2020.113667

[CR10] Esser A, Ziegler P, Kaifie A, Kraus T, Schettgen T (2021b) Modelling past human internal exposure to lower chlorinated indicator PCBs using proxies - a calculation based on multiple longitudinal PCB analyses. Sci Total Environ 784:147250. 10.1016/j.scitotenv.2021.147250 34088037 10.1016/j.scitotenv.2021.147250

[CR11] Gabrio T, Piechotowski I, Wallenhorst T et al (2000) PCB-blood levels in teachers, working in PCB-contaminated schools. Chemosphere 40(9–11):1055–1062. 10.1016/s0045-6535(99)00353-7 10739046 10.1016/s0045-6535(99)00353-7

[CR12] Grimm FA, Hu D, Kania-Korwel I et al (2015) Metabolism and metabolites of polychlorinated biphenyls. Crit Rev Toxicol 45(3):245–272. 10.3109/10408444.2014.999365 25629923 10.3109/10408444.2014.999365 PMC4383295

[CR13] Hannah TJ, Megson D, Sandau CD (2022) A review of the mechanisms of by-product PCB formation in pigments, dyes and paints. Sci Total Environ 852:158529. 10.1016/j.scitotenv.2022.158529 36063921 10.1016/j.scitotenv.2022.158529

[CR14] Hashmi MZ, Khan KY, Hu J et al (2015) Hormetic effects of noncoplanar PCB exposed to human lung fibroblast cells (HELF) and possible role of oxidative stress. Environ Toxicol 30(12):1385–1392. 10.1002/tox.22008 24942145 10.1002/tox.22008

[CR15] Idda T, Bonas C, Hoffmann J et al (2020) Metabolic activation and toxicological evaluation of polychlorinated biphenyls in *Drosophila melanogaster*. Sci Rep 10(1):21587. 10.1038/s41598-020-78405-z 33299007 10.1038/s41598-020-78405-z PMC7726022

[CR16] JEFCA (2016) Non-dioxin-like polychlorinated biphenyls. Joint FAO/WHO Expert Committee on Food Additives (WHO Food Additives Series: 71 S1):431pp

[CR17] Kraus T, Gube M, Lang J et al (2012) Surveillance program for former PCB-exposed workers of a transformer and capacitor recycling company, family members, employees of surrounding companies, and area residents–executive summary. J Toxicol Environ Health A 75(19–20):1241–1247. 10.1080/15287394.2012.709377 22994578 10.1080/15287394.2012.709377

[CR18] Laden F, Ishibe N, Hankinson SE et al (2002) Polychlorinated biphenyls, cytochrome P450 1A1, and breast cancer risk in the Nurses’ Health Study. Cancer Epidemiol Biomark Prev 11(12):1560–1565 12496044

[CR19] Lauby-Secretan B, Loomis D, Baan R et al (2016) Use of mechanistic data in the IARC evaluations of the carcinogenicity of polychlorinated biphenyls and related compounds. Environ Sci Pollut Res Int 23(3):2220–2229. 10.1007/s11356-015-4829-4 26077316 10.1007/s11356-015-4829-4

[CR20] Liu X, Mullin MR, Egeghy P et al (2022) Inadvertently generated PCBs in consumer products: concentrations, fate and transport, and preliminary exposure assessment. Environ Sci Technol 56(17):12228–12236. 10.1021/acs.est.2c02517 35943277 10.1021/acs.est.2c02517 PMC9511961

[CR21] Liu Y, Hu K, Jia H, Jin G, Glatt H, Jiang H (2017) Potent mutagenicity of some non-planar tri- and tetrachlorinated biphenyls in mammalian cells, human CYP2E1 being a major activating enzyme. Arch Toxicol 91(7):2663–2676. 10.1007/s00204-016-1904-7 27913846 10.1007/s00204-016-1904-7

[CR22] Ludewig G, Lehmann L, Esch H, Robertson LW (2008) Metabolic activation of PCBs to carcinogens in vivo - a review. Environ Toxicol Pharmacol 25(2):241–246. 10.1016/j.etap.2007.10.029 18452002 10.1016/j.etap.2007.10.029 PMC2364599

[CR23] Marabini L, Calo R, Fucile S (2011) Genotoxic effects of polychlorinated biphenyls (PCB 153, 138, 101, 118) in a fish cell line (RTG-2). Toxicol in Vitro 25(5):1045–1052. 10.1016/j.tiv.2011.04.004 21504788 10.1016/j.tiv.2011.04.004

[CR24] Marek RF, Thorne PS, Herkert NJ, Awad AM, Hornbuckle KC (2017) Airborne PCBs and OH-PCBs inside and outside urban and rural U.S. schools. Environ Sci Technol 51(14):7853–7860. 10.1021/acs.est.7b01910 28656752 10.1021/acs.est.7b01910 PMC5777175

[CR25] Matsusue K, Ariyoshi N, Oguri K, Koga N, Yoshimura H (1996) Role of cytochrome b5 in the oxidative metabolism of polychlorinated biphenyls catalyzed by cytochrome P450. Xenobiotica 26(4):405–414. 10.3109/00498259609046719 9173681 10.3109/00498259609046719

[CR26] McGraw JE Sr, Waller DP (2006) Specific human CYP 450 isoform metabolism of a pentachlorobiphenyl (PCB-IUPAC# 101). Biochem Biophys Res Commun 344(1):129–133. 10.1016/j.bbrc.2006.03.122 16616008 10.1016/j.bbrc.2006.03.122

[CR27] Moysich KB, Shields PG, Freudenheim JL et al (1999) Polychlorinated biphenyls, cytochrome P4501A1 polymorphism, and postmenopausal breast cancer risk. Cancer Epidemiol Biomark Prev 8(1):41–44 9950238

[CR28] Nelson DR, Koymans L, Kamataki T et al (1996) P450 superfamily: update on new sequences, gene mapping, accession numbers and nomenclature. Pharmacogenetics 6(1):1–42. 10.1097/00008571-199602000-00002 8845856 10.1097/00008571-199602000-00002

[CR29] Nowell S, Sweeney C, Hammons G, Kadlubar FF, Lang NP (2002) CYP2A6 activity determined by caffeine phenotyping: association with colorectal cancer risk. Cancer Epidemiol Biomark Prev 11(4):377–383 11927498

[CR30] Parkinson A, Safe SH, Robertson LW et al (1983) Immunochemical quantitation of cytochrome P-450 isozymes and epoxide hydrolase in liver microsomes from polychlorinated or polybrominated biphenyl-treated rats. A study of structure-activity relationships. J Biol Chem 258(9):5967–5976 6304102

[CR31] Quinete N, Esser A, Kraus T, Schettgen T (2017) PCB 28 metabolites elimination kinetics in human plasma on a real case scenario: study of hydroxylated polychlorinated biphenyl (OH-PCB) metabolites of PCB 28 in a highly exposed German Cohort. Toxicol Lett 276:100–107. 10.1016/j.toxlet.2017.05.025 28552772 10.1016/j.toxlet.2017.05.025

[CR32] Quinete N, Kraus T, Belov VN, Aretz C, Esser A, Schettgen T (2015) Fast determination of hydroxylated polychlorinated biphenyls in human plasma by online solid phase extraction coupled to liquid chromatography-tandem mass spectrometry. Anal Chim Acta 888:94–102. 10.1016/j.aca.2015.06.041 26320963 10.1016/j.aca.2015.06.041

[CR33] Randerath I, Quinete N, Muller JP et al (2024) Partial dechlorination of 2,4,4′-trichlorobiphenyl (PCB 28) mediated by recombinant human CYP1A2. Arch Toxicol 98(1):159–163. 10.1007/s00204-023-03621-1 37917334 10.1007/s00204-023-03621-1 PMC10761437

[CR34] Registry AfTSaD (2000) Toxicological profile for polychlorinated biphenyls (PCBs) 36888731

[CR35] Rengelshausen J, Randerath I, Schettgen T et al (2023) Ten years after: findings from the medical surveillance program on Health Effects in High-Level Exposure to PCB (HELPcB). Arch Toxicol 97(10):2609–2623. 10.1007/s00204-023-03578-1 37594590 10.1007/s00204-023-03578-1 PMC10474999

[CR36] Safe SH (1994) Polychlorinated biphenyls (PCBs): environmental impact, biochemical and toxic responses, and implications for risk assessment. Crit Rev Toxicol 24(2):87–149. 10.3109/10408449409049308 8037844 10.3109/10408449409049308

[CR37] Sargent L, Roloff B, Meisner L (1989) In vitro chromosome damage due to PCB interactions. Mutat Res 224(1):79–88. 10.1016/0165-1218(89)90006-2 2505070 10.1016/0165-1218(89)90006-2

[CR38] Sargent LM, Sattler GL, Roloff B et al (1992) Ploidy and specific karyotypic changes during promotion with phenobarbital, 2,5,2′,5′-tetrachlorobiphenyl, and/or 3,4,3′4′-tetrachlorobiphenyl in rat liver. Cancer Res 52(4):955–962 1737357

[CR39] Schettgen T, Alt A, Preim D, Keller D, Kraus T (2012a) Biological monitoring of indoor-exposure to dioxin-like and non-dioxin-like polychlorinated biphenyls (PCB) in a public building. Toxicol Lett 213(1):116–121. 10.1016/j.toxlet.2011.06.005 21699966 10.1016/j.toxlet.2011.06.005

[CR40] Schettgen T, Gube M, Esser A, Alt A, Kraus T (2012b) Plasma polychlorinated biphenyls (PCB) levels of workers in a transformer recycling company, their family members, and employees of surrounding companies. J Toxicol Environ Health A 75(8–10):414–422. 10.1080/15287394.2012.674905 22686300 10.1080/15287394.2012.674905

[CR41] Shimada T, Imai Y, Sato R (1981) Covalent binding of polychlorinated biphenyls to proteins by reconstituted monooxygenase system containing cytochrome P-450. Chem Biol Interact 38(1):29–44. 10.1016/0009-2797(81)90151-4 6799213 10.1016/0009-2797(81)90151-4

[CR42] Shimada T, Kakimoto K, Takenaka S et al (2016) Roles of human CYP2A6 and monkey CYP2A24 and 2A26 cytochrome P450 enzymes in the oxidation of 2,5,2′,5′-tetrachlorobiphenyl. Drug Metab Dispos 44(12):1899–1909. 10.1124/dmd.116.072991 27625140 10.1124/dmd.116.072991 PMC6047209

[CR43] Srinivasan A, Lehmler HJ, Robertson LW, Ludewig G (2001) Production of DNA strand breaks in vitro and reactive oxygen species in vitro and in HL-60 cells by PCB metabolites. Toxicol Sci 60(1):92–102. 10.1093/toxsci/60.1.92 11222876 10.1093/toxsci/60.1.92

[CR44] Stadnicki SS, Lin FS, Allen JR (1979) DNA single strand breaks caused by 2,2′,5,5′-tetrachlorobiphenyl and its metabolites. Res Commun Chem Pathol Pharmacol 24(2):313–327 111326

[CR45] Sun P, Basu I, Hites RA (2006) Temporal trends of polychlorinated biphenyls in precipitation and air at chicago. Environ Sci Technol 40(4):1178–1183. 10.1021/es051725b 16572772 10.1021/es051725b

[CR46] Tanner JA, Tyndale RF (2017) Variation in CYP2A6 activity and personalized medicine. J Pers Med. 10.3390/jpm7040018 29194389 10.3390/jpm7040018 PMC5748630

[CR47] Uwimana E, Ruiz P, Li X, Lehmler HJ (2019) Human CYP2A6, CYP2B6, AND CYP2E1 atropselectively metabolize polychlorinated biphenyls to hydroxylated metabolites. Environ Sci Technol 53(4):2114–2123. 10.1021/acs.est.8b05250 30576102 10.1021/acs.est.8b05250 PMC6380921

[CR48] Vasko T, Hoffmann J, Gostek S et al (2018) Telomerase gene expression bioassays indicate metabolic activation of genotoxic lower chlorinated polychlorinated biphenyls. Sci Rep 8(1):16903. 10.1038/s41598-018-35043-w 30443001 10.1038/s41598-018-35043-w PMC6237825

[CR49] Warner NA, Martin JW, Wong CS (2009) Chiral polychlorinated biphenyls are biotransformed enantioselectively by mammalian cytochrome P-450 isozymes to form hydroxylated metabolites. Environ Sci Technol 43(1):114–121. 10.1021/es802237u 19209593 10.1021/es802237u

[CR50] Wyndham C, Devenish J, Safe S (1976) The in vitro metabolism, macromolecular binding and bacterial mutagenicity of 4-chloribiphenyl, a model PCB substrate. Res Commun Chem Pathol Pharmacol 15(3):563–570 825937

[CR51] Zanger UM, Schwab M (2013) Cytochrome P450 enzymes in drug metabolism: regulation of gene expression, enzyme activities, and impact of genetic variation. Pharmacol Ther 138(1):103–141. 10.1016/j.pharmthera.2012.12.007 23333322 10.1016/j.pharmthera.2012.12.007

[CR52] Zhang Y, Wise JP, Holford TR et al (2004) Serum polychlorinated biphenyls, cytochrome P-450 1A1 polymorphisms, and risk of breast cancer in Connecticut women. Am J Epidemiol 160(12):1177–1183. 10.1093/aje/kwh346 15583370 10.1093/aje/kwh346

[CR53] Zhao HX, Adamcakova-Dodd A, Hu D et al (2010) Development of a synthetic PCB mixture resembling the average polychlorinated biphenyl profile in Chicago air. Environ Int 36(8):819–827. 10.1016/j.envint.2009.03.003 19375801 10.1016/j.envint.2009.03.003 PMC2888912

