# Supplementary material for: Metabolic activation of WHO-congeners PCB28, 52, and 101 by human CYP2A6: evidence from in vitro and in vivo experiments
Source: Arch Toxicol. 2024 Aug 13;98(11):3739–53. doi: 10.1007/s00204-024-03836-w (PMC11489226; doi:10.1007/s00204-024-03836-w)
Supplement: Supplementary file 1 — Supplementary file1 (DOCX 346 KB) [file 204_2024_3836_MOESM1_ESM.docx]

Supplemental material to:

**Metabolic activation of WHO-congeners PCB28, 52, and 101 by Human CYP2A6: Evidence from In Vitro and In Vivo Experiments**

Isabella Randerath^1^, Thomas Schettgen^1^, Julian Peter Müller^2^, Jens Rengelshausen^1^, Susanne Ziegler^3^, Nathalia Quinete^4^, Jens Bertram^1^, Salah Laieb^1^, Elke Schaeffeler^5^, Andrea Kaifie^1^ , Katja S. Just^2^, Aaron Voigt^6^, Roman Tremmel^5^, Matthias Schwab^5,7^, Julia C. Stingl^2^, Thomas Kraus^1^ and Patrick Ziegler^1#^

1. Institute for Occupational, Social and Environmental Medicine, Medical Faculty, RWTH Aachen University, Pauwelsstrasse 30, 52074, Aachen, Germany

2. Institute of Clinical Pharmacology, University Hospital of RWTH, 52074, Aachen, Germany

3. Institute of Experimental Medicine and Systems Biology, RWTH Aachen University, Aachen, Germany.

4. Department of Chemistry and Biochemistry, Institute of Environment, Florida International University, 3000 NE 151st Street, North Miami, Florida, 33181, USA

5. Dr. Margarete Fischer-Bosch-Institute of Clinical Pharmacology, Stuttgart and University of Tuebingen, Tuebingen, Germany

6. Department of Neurology, University Medical Center, RWTH Aachen University, 52074, Aachen, Germany.

7. Departments of Clinical Pharmacology, and Pharmacy and Biochemistry, University of Tuebingen, Tuebingen, Germany

# Correspondence to: pziegler@ukaachen.de

**Supplemental figure and tables:**


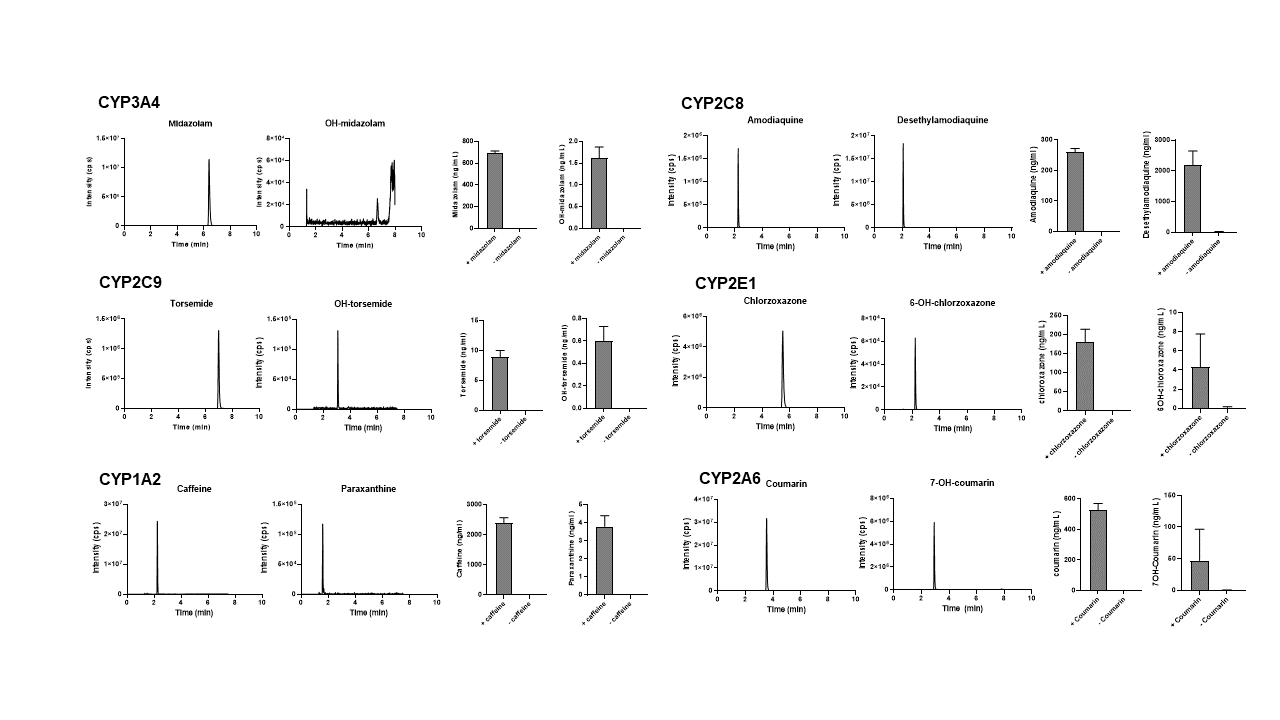


Supplemental figure S1: Metabolism of caffeine, amodiaquine, torasemide, midazolam, chloroxazone and coumarin in **transgenic HEK293 cells stably expressing human CYP-enzymes*.*** Cell lines harboring CYP1A2, CYP2C8, CYP2E1, CYP2A6, CYP3A4 and CYP2C9 were incubated with 10 µM of CYP-enzyme specific substrate for 24 hours with subsequent collection of the medium supernatant and methanol cell lysis. Metabolite production was assessed by LC/MS. Representative chromatograms are depicted. Results show mean +/- SD for three different experiments.


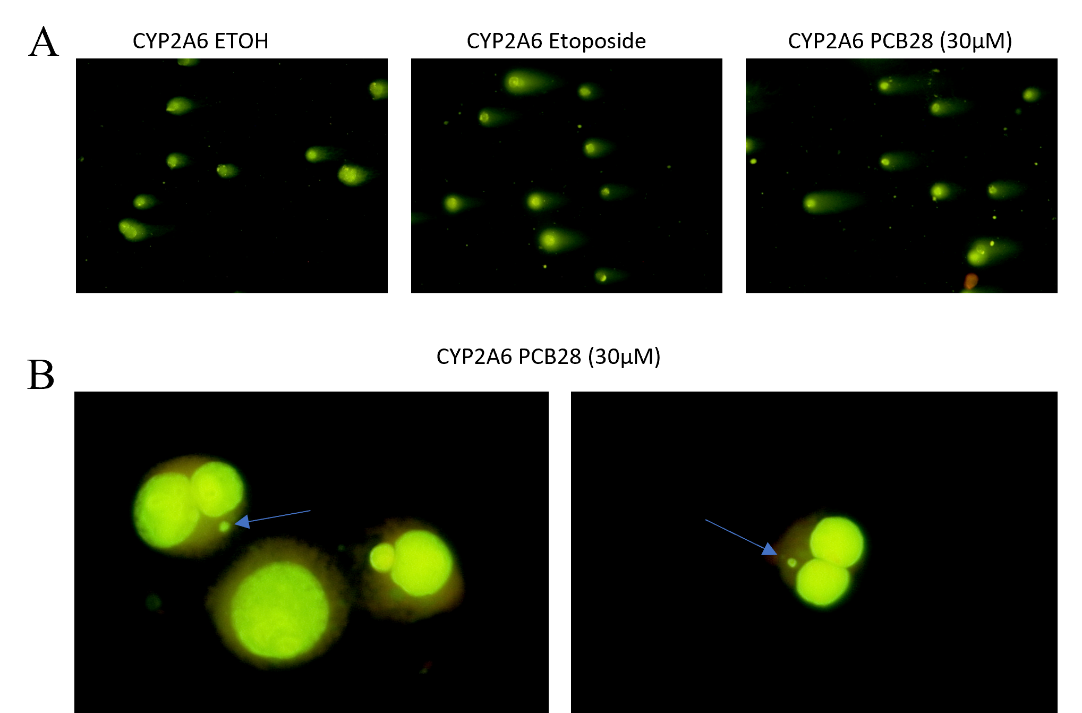


Supplemental figure 2: A) Respective analysis of comet assay in CYP2A6 cells subjected to ethanol (negative control), etoposide (positive control) and PCB28. Samples were examined under a fluorescence microscope (Leica DM6000B with filter I3 450-490nm), and images were taken of at least 50 cells, which were subsequently analyzed. B) Respective analysis of micronucleus formation in CYP2A6 cells subjected to PCB28. Only cells that are binucleated and contain a micronucleus ranging in size from 1/16 to 1/3 of the nucleus, which is visually distinct from the nuclei, were counted as micronucleated. Moreover, only cells with exactly one micronucleus were included, while nucleoplasmic bridges (NPBs) and nuclear buds (BUDs) were not considered.

Supplemental figure 3: Matrix effect. Due to ubiquitous presence of xanthines in human urine, the matrix effect was assessed by comparing the slope of calibration curves constructed in urine diluent and in blank matrix (water).

| **Metabolite** | **5-OHCB28** | **4-OHCB31** | **3´-OHCB28/**  **4´-OHCB25** | **3-OHCB52** | **4-OHCB52** | **3´-OHCB101** | **4´-OHCB101** |
| --- | --- | --- | --- | --- | --- | --- | --- |
| **RT** | 19,14 | 17,93 | 18,66 | 17,12 | 17,98 | 20,2 | 20,77 |
| **LOQ** | 0,02 | 0,05 | 0,02 | 0,02 | 0,02 | 0,05 | 0,05 |
| CYP1A2, clone G5 | 6,22 | 0,69 | 0,56 | ND | ND | ND | ND |
| CYP1A2, clone G8 | 9,07 | 1,11 | 0,84 | ND | ND | ND | ND |
| CYP2C8  Clone A3 | <LOQ | <LOQ | <LOQ | <LOQ | <LOQ | ND | ND |
| CYP2C8  Clone D8 | <LOQ | <LOQ | <LOQ | 0,09 | 0,1 | ND | ND |
| CYP2C8  Clone D9 | <LOQ | <LOQ | <LOQ | <LOQ | <LOQ | ND | ND |
| CYP2E1  Clone B4 | <LOQ | <LOQ | <LOQ | 0,38 | 5,55 | <LOQ | 3,84 |
| CYP2E1  Clone B5 | <LOQ | <LOQ | <LOQ | 0,59 | 8,9 | <LOQ | 4,02 |
| CYP2E1  Clone E6 | <LOQ | <LOQ | <LOQ | 0,74 | 10,23 | <LOQ | 5,08 |
| CYP2A6  Clone B7 | 1,84 | 1,1 | 0,301 | 0,87 | 319,55 | 39,4 | 441,30 |
| CYP2A6  Clone C4 | 1,33 | 0,63 | 0,171 | 0,75 | 1029,16 | 24,09 | 272,55 |
| CYP2A6  Clone C5 | 0,22 | 0,11 | 0,031 | ND | 30,78 | 4,2 | 55,93 |
| CYP3A4  Clone B4 | <LOQ | <LOQ | <LOQ | 0,21 | 0,23 | ND | ND |
| CYP3A4  Clone D10 | <LOQ | <LOQ | <LOQ | 0,66 | 0,72 | <LOQ | <LOQ |
| CYP3A4  Clone E6 | <LOQ | <LOQ | <LOQ | 0,45 | 0,54 | <LOQ | <LOQ |
| CYP2C9  Clone B4 | ND | ND | ND | ND | ND | ND | ND |
| CYP2C9  Clone B5 | ND | ND | ND | ND | ND | ND | ND |
| CYP2C9  Clone C7 | ND | ND | ND | ND | ND | ND | ND |

Supplemental table 1: Quantification of metabolites generated from PCB28, 52, and 101 in different transgenic cell clones, depicted in nM. RT= retention time; <LOQ = below the limit of quantification; ND= not detected.

| **ID** | **PCB28**  **[µg/l]** | **PCB52**  **[µg/l]** | **PCB101**  **[µg/l]** | **PCB153**  **[µg/l]** | **PCB138**  **[µg/l]** | **PCB180**  **[µg/l]** |
| --- | --- | --- | --- | --- | --- | --- |
| AA | 0.1 | 0.005 | 0.005 | 0.59 | 0.358 | 0.377 |
| AB | 0.309 | 0.043 | 0.227 | 15.630 | 14.883 | 8.852 |
| AC | 0.21 | 0.031 | 0.111 | 1.765 | 1.886 | 0.951 |
| AD | 0.823 | 0.026 | 0.073 | 1.397 | 0.984 | 1.158 |
| AE | 1.48 | 0.005 | 0.005 | 1.227 | 0.773 | 1.278 |
| AF | 2.687 | 0.348 | 0.519 | 1.907 | 2.083 | 0.998 |
| AG | 0.019 | 0.005 | 0.005 | 0.945 | 0.718 | 0.612 |
| AH | 0.999 | 0.04 | 0.078 | 1.254 | 1.047 | 0.95 |
| AI | 0.023 | 0.005 | 0.005 | 0.855 | 0.610 | 0.508 |
| AJ | 0.051 | 0.021 | 0.030 | 1.081 | 0.738 | 0.934 |

Supplemental table 2: Concentrations of the WHO congeners PCB28, PCB52, PCB101, PCB153, PCB138 and PCB180 from HELPcB participants in the CYP phenotyping cocktail study determined in 2010.

|  | Interday | | | | Intraday | | | |
| --- | --- | --- | --- | --- | --- | --- | --- | --- |
| Conc. (ng/ml) | Paraxanthine | | 1,7-DMU | | Paraxanthine | | 1,7-DMU | |
|  | % CV | % Acc. | % CV | % Acc. | % CV | % Acc. | % CV | % Acc. |
| 10 | 5.95 | 102.31 | 6.68 | 92.76 | 3.34 | 99.21 | 5.8 | 95.42 |
| 50 | 0.69 | 100.3 | 3.1 | 92.58 | 1.21 | 101.14 | 1.66 | 93.03 |
| 200 | 0.44 | 100.5 | 1.94 | 97.18 | 1.79 | 101.2 | 3.15 | 93.51 |
| 8000 | 2.06 | 99.37 | 2.97 | 95.65 | 1.09 | 95.98 | 4.04 | 92.19 |
| 20000 | 1.38 | 94.65 | 4.5 | 91.8 | 1.44 | 93.17 | 2.7 | 90.3 |

Supplemental table 3. Inter- and intraday accuracy and precision. For interday accuracy and precision three sets of calibrators and quality control samples were processed and measured separately over three days. The average accuracy and precision for the quality control samples over the three batches are given. For intraday accuracy and precision, one set of calibrators and n=5 quality control samples at each level were processed and measured in a single batch. The average accuracy and precision for the quality control samples are given. 1,7-DMU: 1,7-dimethyluric acid; Conc.: concentration; CV: coefficient of variation; Acc.: accuracy

|  | Room temp stability | | | | Freeze-thaw stability | | | | Autosampler stability | | | |
| --- | --- | --- | --- | --- | --- | --- | --- | --- | --- | --- | --- | --- |
| Conc. (ng/ml) | Paraxanthine | | 1,7-DMU | | Paraxanthine | | 1,7-DMU | | Paraxanthine | | 1,7-DMU | |
|  | % CV | % Acc. | % CV | % Acc. | % CV | % Acc. | % CV | % Acc. | % CV | % Acc. | % CV | % Acc. |
| 10 | 3.62 | 107.47 | 2.73 | 86.89 | 1.44 | 102.81 | 6.39 | 92.35 | 2.69 | 91.07 | 6.92 | 87.73 |
| 50 | 1.23 | 100.1 | 0.82 | 93.57 | 1.08 | 100.21 | 1.07 | 93.82 | 1.44 | 96.04 | 11.24 | 91.91 |
| 200 | 1.23 | 97.57 | 0.7 | 94.73 | 1.04 | 98.52 | 4.28 | 95.77 | 1.39 | 95.81 | 2.29 | 97.17 |
| 8000 | 1.13 | 95.75 | 2.27 | 92.99 | 0.59 | 95.06 | 1.66 | 91.84 | 0.16 | 95.54 | 1.46 | 90.92 |
| 20000 | 0.77 | 91.73 | 1.05 | 88.08 | 1.67 | 90.49 | 0.8 | 86.71 | 1.82 | 93.8 | 1.86 | 88.23 |

Supplemental table 4. Assessment of short-term stability. Room temp stability: unprocessed quality control samples (n=3) were shelved at room temperature for 24 hours and were then processed and measured against a freshly prepared set of calibrators and quality control samples. Freeze-thaw stability: quality control samples (n=3) were thawed and refrozen three-times over separate days and were measured against a freshly prepared set of calibrators and quality control samples. Autosampler stability: quality control samples (n=3) were processed and stored in the autosampler for 12 hours at 4°C and were measured against a freshly prepared set of calibrators and quality control samples. Room temp.: room temperature; 1,7-DMU: 1,7-dimethyluric acid; Conc.: concentration; CV: coefficient of variation; Acc.: accuracy

| Dilution factor | Paraxanthine | | 1,7-DMU | |
| --- | --- | --- | --- | --- |
|  | % CV | % Acc. | % CV | % Acc. |
| 1 to 50 | 2.76 | 100.75 | 1.17 | 96.23 |
| 1 to 10 | 1.42 | 100.23 | 1.68 | 94.22 |
| 1 to 5 | 0.81 | 95.06 | 0.94 | 89.58 |
|  |  |  |  |  |

Supplemental table 5: Dilution integrity in urine diluent. Urine diluent was spiked with 100.000 ng/ml of paraxanthine and 1,7-dimethyluric acid and was diluted further with urine diluent at 1/5, 1/10 and 1/50. For each level of dilution n=5 samples were prepared. Diluted samples were processed together with a fresh set of calibrators and quality control samples.1,7-DMU: 1,7-dimethyluric acid; CV: coefficient of variation; Acc.: accuracy

|  | Conc. (ng/ml) | Paraxanthine | | 1,7-DMU | |
| --- | --- | --- | --- | --- | --- |
|  |  | % CV | % Acc. | % CV | % Acc. |
| Calibrators | 10 | 3.1 | 100 | 1.87 | 98.58 |
|  | 50 | 2.89 | 99.15 | 3.84 | 103.08 |
|  | 100 | 2.2 | 101.4 | 3.18 | 107.64 |
|  | 1000 | 1.39 | 102.1 | 4.74 | 104.81 |
|  | 5000 | 1.67 | 102.49 | 2.53 | 100.92 |
|  | 10000 | 1.48 | 99.02 | 1.73 | 95.75 |
|  | 25000 | 1.81 | 95.82 | 2.99 | 89.23 |
| Quality Controls | 10 | 7.23 | 101.02 | 7.23 | 87.96 |
|  | 50 | 4.37 | 100.4 | 5.28 | 94.68 |
|  | 200 | 2.19 | 98.16 | 3.78 | 99.7 |
|  | 8000 | 1.42 | 100.2 | 3.15 | 94.25 |
|  | 20000 | 3.61 | 97.35 | 4.6 | 89.32 |

Supplemental table 6: Average accuracy and precision over all measured study sample batches. Included were 4 batches with 4 sets of calibrators and quality controls each, which were injected twice per each run (n=8 values). 1,7-DMU: 1,7-dimethyluric acid; Conc.: concentration; CV: coefficient of variation; Acc.: accuracy

**Supplemental material and methods:**

The following probe substrates and analytical standards have been used in this study:

Amodiaquine (1031004-500MG, Sigma-Aldrich), desethylamodiaquine (D-039-1ML, Supelco), midazolam (M908, Sigma-Aldrich), 1-hydroxymidazolam (Cay10385-5, Cayman Chemical), torsemide (HY-B0247, MedChemExpress), hydroxytorsemide (sc-394035, Santa cruz), caffeine (C0750-100G, Sigma-Aldrich), paraxanthine (D5385-100MG, Sigma-Aldrich), coumarin (C4261-50G, Sigma- Aldrich), 7-hydroxycoumarin (121110250, Thermo-Scientific), chlorzoxazone (C4397-25G, Sigma-Aldrich), 6-hydroxychlorzoxazone (UC148, Merck).

**Incubation of cells:**

HEK293 cells overexpressing the respective CYP enzymes were seeded onto 6-well plates and were treated with or without 10 µM of the respective probe drug in DMEM medium for 24 hours at 37 °C and 5 % CO_2_. After incubation 200 µL DMEM were collected, cells were washed with PBS and adherent cells were lysed in 500 µL methanol for 20 minutes. Analytes were measured by LC-MS/MS either in methanol cell lysates or in DMEM supernatant. DMEM: midazolam/1-OH-midazolam, caffeine/paraxanthine, amodiaquine/desethylamodiaquine, chlorzoxazone/6-OH-chlorzoxazone, coumarin/7-OH-coumarin; methanol cell lysates: torsemide/OH-torsemide

**Sample preparation and LC-MS analysis:**

50 µL of DMEM samples were protein precipitated by adding 200 µL of methanol and were centrifuged for 20 min, 17,000 *g* and 4°C. Supernatants were diluted in water or 40 % methanol according to dilution factor. Methanol cell lysates were diluted ½ with water and centrifuged for 20 min, 17,000 g and 4°C. 5 µL of the processed samples were injected for LC-MS analysis. Samples were measured on an Agilent 1290 Infinity II UHPLC coupled to a SCIEX QTRAP6500+ triple quadrupole mass spectrometer.

Analytes were separated on an Agilent Poroshell 120 EC-C18 column (1.9 µm, 2.1 x 50 mm; 699675‑902, Agilent Technologies) with A: 0.1 % formic acid in water and B: methanol. Analyte separation was performed with a gradient of gradual changes in % B. The gradient was as follows: 5 % B at 0 min, 15 % B at 1 min, 25 % B at 2.5 min, 34 % B at 7 min, 95 % B at 7.5 min, 95 % B at 8.5 min, 5 % B at 8.7 min held to 10 min.

Analytes were measured in a single multiplexed LC-MS method with multiple reaction monitoring (MRM) in positive and negative ion mode. Positive ion mode: Amodiaquine 356 🡪 283, 358 🡪 285, desethylamodiaquine 328 🡪 283 and 328 🡪 255, midazolam 326 🡪 291, 326 🡪 249, 1-hydroxymidazolam 342 🡪 324, 342 🡪 203, torsemide 349 🡪 264, 349 🡪 168, hydroxytorsemide 365 🡪 280, 365 🡪 306, caffeine 195 🡪 138, 195 🡪 110, paraxanthine 181 🡪 124, 181 🡪 96, coumarin 147 🡪 103, 147 🡪 91, 7-hydroxycoumarin 163 🡪 107, 163 🡪 91. Negative ion mode: chlorzoxazone 168 🡪 132, 168 🡪 35, 6-hydroxychlorzoxazone 184 🡪 120, 184 🡪 64

**Validation of the LC-MS quantification method for 1,7-dimethyluric acid and paraxanthine in human urine**

Paraxanthine and 1,7-dimethyluric acid stocks for calibrators and quality controls were weighed in separately. Stock solutions were prepared in DMSO. Calibration curves in urine diluent consisted of 7 non-zero calibrators with nominal values of 10, 50, 100, 1000, 5000, 10000 and 25000 ng/mL and were weighted with 1/x^2^. Quality controls at 5 different levels were used with nominal values of 10, 50, 200, 8000 and 20000 ng/mL.

The analytical method was validated by assessing interday and intraday accuracy and precision, short-term stability (benchtop stability at room-temperature, freeze-thaw stability and autosampler stability), long-term stability, dilution integrity and matrix effect. An accuracy and precision (CV) of ± 15 % for all calibrators and quality control samples was achieved in all validation assays.

**Long-term stability**

Stock solutions and aliquoted calibrator and quality control samples were stored at -32°C. Freshly weighed standards were spiked into urine diluent at low (50 ng/mL) and high (20000 ng/mL) concentrations and were compared to shelved aliquots with the same concentration. Both analytes were stable in urine diluent for at least 43 days.
